# Single-cell RNA reveals a tumorigenic microenvironment in the interface zone of human breast tumors

**DOI:** 10.1186/s13058-023-01703-7

**Published:** 2023-08-29

**Authors:** Wei Yang, Meiyu Xu, Shuoqi Xu, Qingxian Guan, Shuaiming Geng, Juanhong Wang, Wei Wei, Hongwei Xu, Ying Liu, Yong Meng, Ming-Qing Gao

**Affiliations:** 1 College of Life Sciences, Northwest University, Xi’an, China; 2 Department of Pathology, Xi’an No.3 Hospital, The Affiliated Hospital of Northwest University, Xi’an, China; 3 Basic Medical College, Qingdao University, Qingdao, China; 4 School of Medicine, Northwest University, Xi’an, China

**Keywords:** Breast cancer, Interface zone, Invasion, Single cell, Heterogeneity

## Abstract

**Background:**

The interface zone, area around invasive carcinoma, can be thought of as the actual tissue of the tumor microenvironment with precedent alterations for tumor invasion. However, the heterogeneity and characteristics of the microenvironment in the interface area have not yet been thoroughly explored.

**Methods:**

For in vitro studies, single-cell RNA sequencing (scRNA-seq) was used to characterize the cells from the tumor zone, the normal zone and the interface zone with 5-mm-wide belts between the tumor invasion front and the normal zone. Through scRNA-seq data analysis, we compared the cell types and their transcriptional characteristics in the different zones. Pseudotime, cell–cell communication and pathway analysis were performed to characterize the zone-specific microenvironment. Cell proliferation, wound healing and clone formation experiments explored the function of differentially expressed gene BMPR1B, which were confirmed by tumor models in vivo.

**Results:**

After screening, 88,548 high-quality cells were obtained and identified. Regulatory T cells, M2 macrophages, angiogenesis-related mast cells, stem cells with weak DNA repair ability, endothelial cells with angiogenic activity, fibroblasts with collagen synthesis and epithelial cells with proliferative activity form a unique tumorigenic microenvironment in the interface zone. Cell–cell communication analysis revealed that there are special ligand–receptor pairs between different cell types in the interface zone, which protects endothelial cell apoptosis and promotes epithelial cell proliferation and migration, compared to the normal zone. Compared with the normal zone, the highly expressed BMPR1B gene promotes the tumorigenic ability of cancer cells in the interface zone.

**Conclusions:**

Our work identified a unique tumorigenic microenvironment of the interface zone and allowed for deeper insights into the tumor microenvironment of breast cancer that will serve as a helpful resource for advancing breast cancer diagnosis and therapy.

**Supplementary Information:**

The online version contains supplementary material available at 10.1186/s13058-023-01703-7.

## Background

Breast cancer has become the most commonly diagnosed cancer in women [1]. In the absence of distant metastases, breast cancer is a potentially curable disease [2]. Breast conserving surgery (BCS) has been favored more recently due to its highest success rate in women with early-stage breast cancer, but it is not recommended for women at high risk of local recurrence [3]. A negative margin reduces the risk of local recurrence in invasive breast cancer based on a study-level meta-analysis [4]. For esthetic reasons, the goal of BCS is to remove a sufficient margin of normal tissue around the tumor without affecting the breast appearance. A 5-mm-wide tissue zone surrounding the tumor burden and adjoining the normal is a traditionally and widely used gross surgical margin showing low recurrence [5]. However, in actual practice, a surgical margin ranges from no tumor on ink to 10 mm or more depending on the age, cancer type, therapies employed and ethnicity [6]. Consequently, for an accurate intraoperative surgical margin assessment of BCS, it is necessary to clarify the cellular, structural and molecular information of the whole specimen surface as it relates to the tumor burden zone.

Tumor microenvironment (TME) plays a decisive role in tumor malignant biological behaviors and response to therapies through its constant interaction with tumor cells [7]. TME is composed of different types of cells, each of which can be classified into different subtypes. For example, fibroblasts include matrix fibroblasts, vascular fibroblasts, cycling fibroblasts and developmental fibroblasts. These fibroblasts have different functions in tumors [8]. T cells mainly include CD4 + T cells, CD8 + T cells and regulatory T cells, each of which has specific states and functions [9]. In addition, TME shows great heterogeneity between the center and the edge of the tumor. The center of the tumor sometimes develops necrosis due to hypoxia, while hypoxic stress at the margin is less [10]. TME exerts distinct pressures in different regions of the tumor, which generates intratumoral heterogeneity [11], and represents a substantial hurdle to precision medicine [12]. Most notably, TME also has different influences on the zones distal to the tumor burden zones, which dictates spatial heterogeneity [13].

Single-cell RNA sequencing (scRNA-seq) techniques can perform sequence analysis of transcripts at a single-cell resolution that reveal changes that render each individual cell type unique [14]. Applications of scRNA-seq techniques in previous studies on lung tumor [15], primary breast cancer [16] and breast cancer metastasis [17] have increased the understanding of cellular diversity found in TME and how the cells interact with each other in complex heterogeneous cancerous tissues.

We have proved that the 5-mm-wide interface zone around breast tumor displayed zone-specific characteristics using a mRNA array [5] and MALDI-MS [18] compared with the normal and tumor zones. In addition, fibroblasts derived from the interface zone had distinct properties such as stronger tumor-promoting abilities than that derived from their counterparts [13, 19]. Based on these findings, we surmised that the interface zone has a special composition and plays an important role in the process of tumor invasion. Here, we applied single-cell RNA sequencing to three zones of breast tissues and characterized the diversity of these zones. We demonstrated that breast tissues of breast cancer patients have dynamic changes that spatially extend from the tumor zone to the distal normal zone of tissue.

## Methods

### Patients and tumor specimens

This study was approved by the Medical Ethics Committee of Northwest University. Patients with primary, highly invasive, non-metastatic breast tumors diagnosed via core biopsy were recruited (Additional file 1: Table S1) and provided signed informed consent. Fresh tumor tissue samples were collected during surgical excision.

Breast specimen was rapidly transported to the research facility after resection in the operating room. An experienced anatomical pathologist grossly examined the tumor positioning and defined three zones in a cutting surface as described in our previous publications [5, 13], including a tumor zone within the tumor boundary, an interface zone within 5 mm of the outer tumor boundary and a distal normal zone at least 10 mm from the outer tumor boundary. For reproducible spatial identification, small invasive adenocarcinoma with an area less than 10 mm^2^, clear tumor boundaries were included in this study. Given that tumors are randomly shaped objects, a breast sample was cut into a series of continuous and parallel sections, and each section had a 3 mm thickness. Only those tissues with three continuous sections with similar tumor boundary in the cutting surface were used (Additional file 1: Figure S1), which can ensure that three zones were precisely defined in a three-dimensional setting. Then, a fraction of tissues from all zones were fixed in formalin and embedded in paraffin for routine histopathological analysis to confirm that the tissue was correctly segmented and that tissues from the interface zone did not contain tumor cells. Finally, tissue pieces were extracted from the remainder of each zone for preparation of single-cell suspension, immunofluorescence examination and bulk RNA sequencing. In particular, tissue pieces derived from the tumor zone were extracted from the edge-most fraction of the tumor to avoid possible tissue necrosis that may be caused by hypoxia.

### Single-cell RNA library preparation and sequencing

Single-cell suspension were prepared by mechanical dissociation and enzymatic digestion after removal of residual blood and surrounding adipose tissues. Cell suspension in which living cells accounted for more than 85% of the total cells was used for construction of single-cell RNA library and sequencing. Briefly, single-cell suspensions were parted into nanoliter-scale Gel Bead-In EMulsions (GEMs) to achieve single-cell resolution according to Chromium Next GEM Single Cell 3ʹ Reagent Kit v3.1 and Chromium Next GEM Chip G Single Cell Kit. Single cells in GEMs were lysed, and RNAs were reverse-transcripted and bar-coded by reverse transcriptase at 53 °C for 45 min and 85 °C for 5 min, and then held at 4 °C. Leftover biochemical reagents and primers in post-GEM reaction mixture were removed by Silane magnetic beads. cDNA was amplified to generate sufficient mass for library construction. Read 1 primer sequences were added during GEM incubation. Read 2 primer, a sample index, P5 and P7 sequences were added by end repair, A tailing, adaptor ligation and PCR during library construction. 16-bp bar code and 12-bp UMI were encoded in Read 1. Read 2 was used to sequence the cDNA fragment. Sample index sequences were incorporated as the i7 index read. Read 1 and Read 2 are standard Illumina® sequencing primer sites used in paired-end sequencing. Sequencing was performed by Illumina Novaseq 6000 in LC-bio Technology Co., Ltd (Hangzhou, Zhejiang, China). All samples were performed with the same process and kits.

### Single-cell RNA-seq data processing

Reads were aligned to genome by STAR software encapsulated in Cell Ranger and further aligned to exons, introns and intergenic regions. These reads aligned to the genome were used for UMI counting. Cells with a percentage of mitochondrial genes below 25% and a gene count exceeding 500 were used for further analysis. After quality control, a total of 88,548 single cells were obtained. Cell data were normalized by LogNormalize. The calculation formula is as follows:

$$ {\text{expression level of A gene }} = {\text{ ln }}\left( {{1 } + \, \left( {{\text{UMI A }} \div {\text{ UMI Total}}} \right) \, \times { 1}0000} \right) $$

Normalized data were used for clustering and visualizing based on their principal component analysis scores by Seurat (version 4.1.0), and a total of 87 clusters were identified. The following criteria were used to identify differential genes: (1) *p* value =  < 0.01; (2) log_2_FC >  = 0.26. log_2_FC means log_2_ fold change; (3) the percentage of cells where genes were detected in a specific cluster more than 10%. A nonparametric manner called gene set variation analysis (GSVA, 1.34.0) was used to estimate hallmark pathway activation for clusters.

### Transcription factor regulon analysis

The analysis of transcription factor (TF) regulon activity was performed by the R package SCENIC (version 1.1.2.2), which infers the gene regulatory network based on co-expression. First, the log-normalized expression matrix was used as the input matrix. Second, co-expression networks of TFs and target genes were inferred by runGenie3 function. Then gene regulatory networks were constructed and scored by the functions of runSCENIC_1_coexNetwork2modules, runSCENIC_2_createRegulons and runSCENIC_3_scoreCells.

### Single-cell trajectory inference analysis

Cell lineage trajectories of macrophages, B cells and stem cells were inferred and characterized at the single-cell level. The UMI count matrix was used as the input. Dimensionality reduction was performed by using DDRTree algorithm based on differentially expressed genes; then, cell lineage trajectories between diverse cell subtypes based on cell cluster and pseudotime were inferred with the default parameters of Monocle (version, 2.22.0). Finally, “plot_genes_in_pseudotime” function was used to visualize dynamic changes of the cell.

### Cell–cell interaction analysis

CellPhoneDB (version, 3.1.0) was used to analyze communications among cell subtypes. First, the average expression level of each gene and the percentage of cells expressing the gene in every cell type were calculated. Then, based on the level of receptors and matched ligands in cell type, the possibility of cell interaction between cell types was inferred. Next, the cell type labels of all cells are randomly arranged 1000 times to calculate the significance of ligand–receptor pairs. Finally, the strength of cellular interactions was evaluated based on the expression of ligand–receptor pairs.

### Gene set signature analysis

Gene set (top 10 marker genes of subcluster) signature was scored by mean value of log_2_(TPM + 1) on TCGA and GTEx data using the GEPIA tool (http://gepia2.cancer-pku.cn/#index). Then, the correlation between prognostic impact and signature was calculated based on the score of gene set signature. The hazards ratio was calculated based on Cox PH Model. Samples were classified as high-signature or low-signature cohorts using percentile cutoffs of 50% or 50%. |log_2_FC|> 1 and *p*-value < 0.01 were set as threshold.

### Immunofluorescence analysis

For immunohistofluorescence staining of breast tissue sections, breast tissues were fixed with 4% paraformaldehyde for 1 day, dehydrated and embedded in paraffin for sections. The sections were dewaxed and rehydrated followed by antigen repair using a sodium citrate water bath at 98 °C for 40 min. After natural cooling, sections were rinsed with PBS 3 times for 5 min each time. Then, using 10% donkey serum at room temperature blocked sections for 1 h followed by incubation with primary antibodies: mouse mAb anti-KRT19 (1:400, Proteintech, #60187–1-lg, RRID:AB_2249705), rabbit pAb anti-IGFBP5 (1:100, Proteintech, #55205–1-AP, RRID:AB_2736835), rabbit pAb anti-DCN (1:400, Proteintech, #14667–1-AP, RRID:AB_2090265) or mouse mAb anti-MMP11 (1:50, Santa Cruz Biotechnology, sc-58381, RRID:AB_2144725) for overnight at 4 °C. After rewarming at 37 °C for 40 min, the sections were cleaned 3 times for 5 min each time. Sections were incubated with fluorochrome-conjugated secondary antibodies at 37 °C for 40 min and cleaned 3 times for 5 min each time. Nuclei were stained by DAPI. Finally, sections were photographed under OLYMPUS inverted fluorescence microscope.

### Cell culture and gene transfection

MCF-7, MDA-MB-231 and SKBR3 human breast cancer cell lines and MCF10A human normal breast epithelial cell line were obtained from Procell Life Science&Technology Co., Ltd. (Procell, Wuhan, China). Short tandem repeat profiling was performed on each cell line. Mycoplasma was tested every two months. Cells were cryopreserved within the first 4 generations after acquisition and used within the 5th generation after each thaw. MCF-7, MDA-MB-231 and MCF10A cell lines were cultured in Procell-recommended media supplemented with 10% FBS (BI). SKBR3 cell line was cultured in RPMI-1640 media (Procell, PM150113) with 13% FBS (BI). Stable BMPR1B gene overexpression cell line was created with lentivirus-mediated gene transfection. Briefly, BMPR1B gene sequence was cloned into lentivirus expression vector (CD531B-1). Next, cell lines were infected with packaged lentivirus for 10 h followed by puromycin treatment. Finally, the level of BMPR1B transcript was detected by real-time quantitative polymerase chain reaction (RT-qPCR).

### RT-qPCR

Total RNA was extracted from each sample using TransZol Up reagent (TransGen, Beijing, China), and reverse transcription was performed to generate cDNA using an EasyScript® One-Step gDNA Removal and cDNA Synthesis SuperMix (TransGen, AE311-02). Real-time PCR was performed using PerfectStart® Green qPCR SuperMix (TransGen, AQ101-01). Primer sequences targeting the bone morphogenetic protein receptor type 1B (BMPR1B) and housekeeping gene of GAPDH are listed in Additional file 1: Table S2. RT-qPCR data were calculated by the ΔΔCt of genes of interest relative to the housekeeping gene GAPDH.

### Cell proliferation assay

Cell proliferation was performed using Cell Counting Kit-8 (TransGene, FC101-01). Cells were plated in 96-well plates and cultured in an incubator. At indicated time point after seeding, CCK-8 solution was added to the wells and incubated for 3 h; then, the absorbance was measured at 450 nm (BioTek, Epoch2 Instruments).

### Colony formation assay

Cells were seeded in 6-well plates at a density of 1000 cells/3 ml per well. The medium was changed every 4 days. On the 6th day (MCF7 cells), 8th day (MDA-MB-231) and 20th day (SKBR3 cells), cell clones were photographed using OLYMPUS inverted fluorescence microscope.

### Wound-healing assay

A thin ruler was used to scrape a straight line in the confluent cell monolayer in 6-well plate to create a wound. Then detached cells were washed away with PBS and the cells were incubated in media with 1% FBS to allow cells to grow and close the wound. The scratches were photographed at 0 h,12 h, 24 h, 48 h, 72 h and 144 h using OLYMPUS inverted fluorescence microscope. The scratch area was calculated by the software OLYMPUS cellSens Entry, and the average value of the area was calculated by multiple scratches. The change rate of area represented the speed of cell migration.

### Tumor-bearing mice

Female BALB/cNj-Foxn1^nu^/Gpt mice were purchased from corporation (Gempharmatech Co., Ltd, China). Mice were housed in specific pathogen-free facilities with ad libitum access to water and food under a 12-h dark and 12-h light cycle in animal housing with constant humidity (40–60%) and temperature (23 ± 1 °C). 5 × 10^6^ cells were collected and injected into subcutaneous location of both groin in nude mice. Cells expressing BMPR1B gene were injected on the left side, and control cells were injected on the right side. All programs have been approved by the Research Ethics Committee of Northwest University.

### Statistical analysis

The log-rank test, Pearson’s correlation and two-sided paired Student’s *t*-test were used in the study. The method for score of gene signatures is one-way ANOVA. Unless otherwise specified, the quantitative data were presented as mean (± standard deviation) from three independent experiments. Significant differences were considered as follows: ns, no significance, *p* >  = 0.05; *p < 0.05; **p < 0.01.

## Results

### scRNA-seq reveals cell subpopulations of different zones of breast tumor tissues

**Fig. 1:**
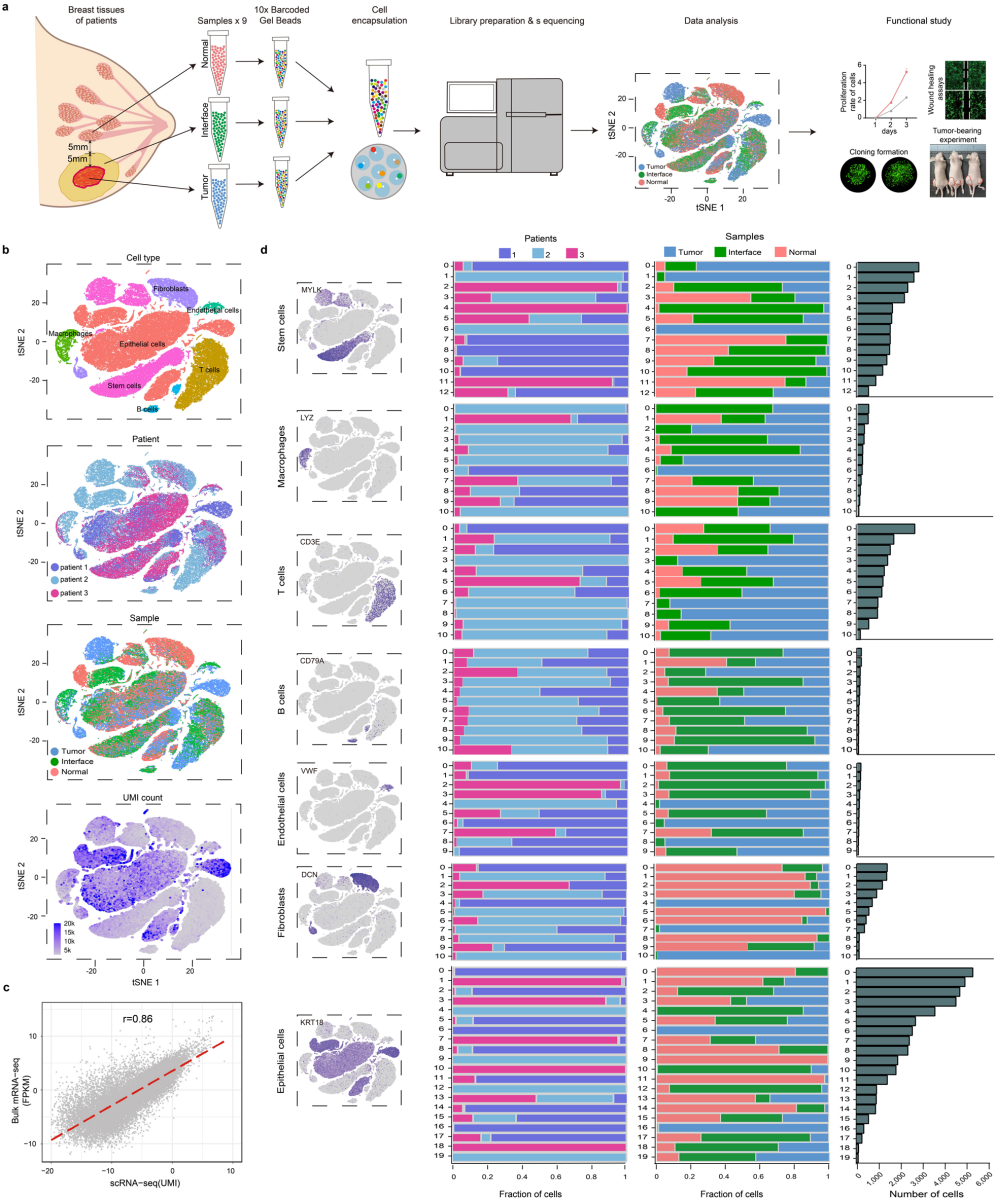
Overview of the 88,548 single cells from breast tissues of breast cancer patients**. a** Design and workflow of this study. **b** tSNE plots of the 88,548 cells profiled (top to bottom): the annotated cell type, different patients of origin (patient 1, patient 2 and patient 3), different sample types of origin (normal, interface or tumor zone samples) and the number of transcripts. K, Thousand. **c** Density dot plot showing the correlation of genes in bulk sequencing and single-cell sequencing. **d** Subclusters of stem cells, macrophages, T cells, B cells, endothelial cells, fibroblasts and epithelial cells (left to right): tSNE plots of marker genes for cell types, the fraction of cells deriving from each of 3 patients, the fraction of cells deriving from the normal, interface and tumor zone samples and the number of cells in each subcluster

We collected nine sets of fresh samples from the tumor zone, the interface zone and the normal zone of 3 patients with untreated and invasive breast cancer who underwent total mastectomy. After resection, samples were rapidly digested to single-cell suspension for sequence, analysis and functional study (Fig. 1a). A total of 88,548 cells were obtained from clean data. Among them, 28,718 cells originated from the tumor zone, 29,192 cells from the interface zone and 30,638 cells from the normal zone (Additional file 1: Table S3). Based on marker genes and artificial correction, all identified clusters could be assigned to known cell types: Fibroblasts, B cells, T cells, epithelial cells, stem cells, macrophages and endothelial cells, and different cell types had different transcriptional activities (Fig. 1b).

Meanwhile, the correlation analysis of single-cell RNA sequencing and matched bulk-seq showed that they had a good correlation (*r* = 0.86; Fig. 1c), indicating that gene expression was not significantly affected by cell dissociation. Overall, the data revealed the presence of a complex cellular heterogeneity containing a total of 87 different cell subclusters (Fig. 1d), and many subclusters had strong zone specificity, which implied that the interface zone has a unique microenvironment. Here, we mainly explored the zone-specific cell subtypes and functions of specific genes in epithelial cells.

### Comparison of characteristics of T cell subtypes in different zones

**Fig. 2:**
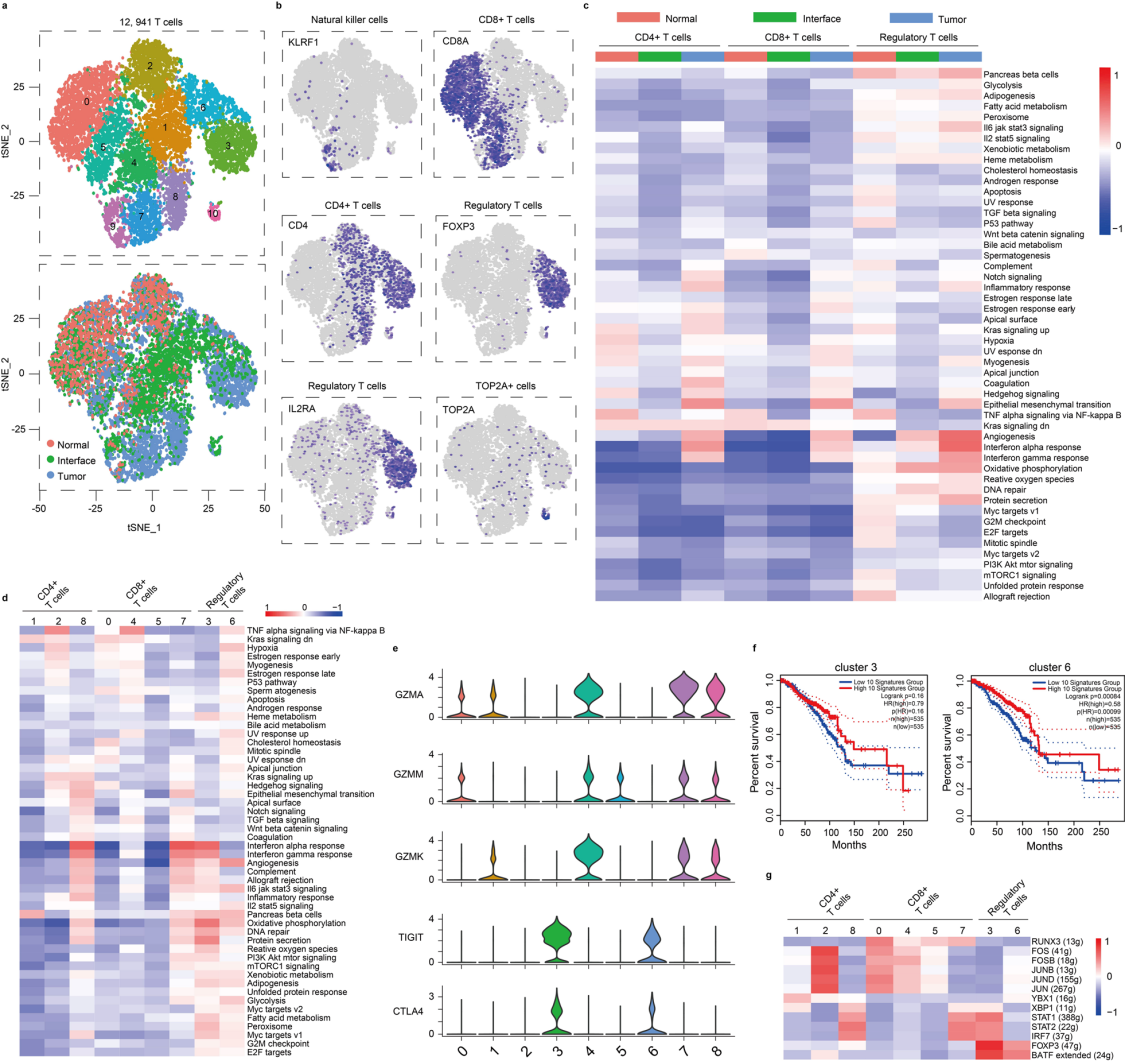
T cell clusters. **a** tSNE plots of 12,941 T cells, color by relevant clusters (top) or sample type (bottom). **b** Color coding for expression (gray to blue) of marker genes of natural killer cells, CD8 + T cells, CD4 + T cells, regulatory T cells and TOP2A + cells. **c** Differences in pathway activity of each cell scored by GSVA between T cells isolated from normal, interface and tumor zone zones. **d** As in **c**, but for CD4 + T cells (clusters 1, 2 and 8), CD8 + T cells (clusters 0, 4, 5 and 7) and regulatory T cells (clusters 3 and 6). **e** Violin plots displaying the expression of selected genes encoding granzyme (GZMA, GZMM and GZMK) and immune checkpoint (CTLA4 and TIGIT) in T cell clusters. **f** Overall survival curves of breast cancer patients stratified by the average expression of top 10 marker genes of regulatory T cell clusters. Binary: high versus low, *n* = 535 or 535 patients, respectively. Expression threshold set to median to split the high-expression and low-expression cohorts. **g** Heatmap of single-cell regulon scores inferred by SCENIC. Number in brackets is the number of the target genes. g, genes

T cells play crucial roles in the process of tumor inflammatory response. Here, we detected 12,941 T cells that were reclustered into 11 separate subsets (Fig. 2a), respectively, corresponding to natural killer cells (NK cells, cluster 9, *KLRF1* +), CD8 + T cells (clusters 0, 4, 5 and 7, *CD8A* +), CD4 + T cells (clusters 1, 2 and 8, *CD4* + */FOXP3-*) and regulatory T cells (Tregs, clusters 3 and 6, *CD4/IL2RA/FOXP3* +) (Fig. 2b). In addition, one cluster did not correspond to known cells (cluster 10, TOP2A +), and ontology analysis showed cell cycle pathway had strong enrichments in this cluster (Additional file 1: Figure S2a), suggesting cluster 10 had an active biological process.

T cell types of NK cells, CD8 + cells and CD4 + cells were detected in all three zones, with the exception of CD8 + cluster 7 and CD4 + cluster 8 mainly presenting in tumor zone. For Tregs, cluster 3 apparently enriched in tumor zone, and cluster 6 enriched in both tumor and interface zone (Fig. 2a and b). The enrichment of specific T cell clusters in tumor and interface zones suggested a transcriptome difference between T cell types that were influenced by microenvironment.

Subsequently, we compared levels of the pathway gene signatures among cells from the three zones (Fig. 2c). Analysis showed that overall pathway signals of CD4 + and CD8 + T cell were weak in the interface zone, which indicated that these T cells have weak bioprocess and functions. However, angiogenesis signal in the interface-derived Tregs was stronger than that in the normal zone, which might contribute to the induction and/or maintenance of Tregs in the interface zone [20]. Besides, some pathways differed between subclusters; for instance, CD8 + cluster 7 and CD4 + cluster 8 showed strong IFN-γ and IFN-α responses, enhanced complement activities, high allograft rejection activities (Fig. 2d) and granzyme expressions of *GZMA*, *GZMM* and *GZMK* (Fig. 2e), indicating these two subclusters of T cells showed some certain anti-tumor activities. For Tregs, compared with cluster 6, cluster 3 had stronger inflammatory response states, such as IFN response and complement reaction (Fig. 2d), although both of them expressed immunosuppressive molecules *TIGIT* and *CTLA4* (Fig. 2e). However, survival analysis of marker genes showed that cluster 6 positively contributed to the prognosis of patients based on The Cancer Genome Atlas (TCGA) datasets, while cluster 3 had no relationship with patients’ prognosis (Fig. 2f), which indicated tregs had complex functions that need to be further explored.

In order to assess which transcription factors underlay differences in expression patterns of pathways among clusters, we performed single-cell regulatory network inference and clustering (SCENIC). SCENIC scanned transcription factor binding sites near differentially expressed gene positions and analyzed co-expression of these genes and transcription factors. There were significant differences in the expression of transcription factors (Fig. 2g). We noticed high expression levels of transcription factors STAT1, STAT2 and IRF7 in tumor-derived cluster 3, cluster 7 and cluster 8 (Fig. 2g). These factors are required for IFN production [21–23], which suggested that they played important roles in inflammatory response. Upregulation of transcription factor BATF controlled the activation program of clusters 3 and 6 in tumor and interface zones [24].

Recently, immune-hot and immune-cold regions are considered additional elements of heterogeneity of TME [25, 26]. Here we found clusters 0, 1, 4, 5, 7 and 8 can express granzyme (Fig. 2e), which suggests that these clusters have immune activity, and thus, they belong to the immune-hot clusters. However, clusters 2, 3 and 6 had no obvious immune activity based on their low granzyme expression level, and they belong to the immune-cold clusters. Notably, we noticed that total cell numbers of immune-hot clusters were higher in the interface or the tumor zone than that in the normal zone in all three patients (Additional file 1: Table S4), indicating the interface zone share some characteristics with the tumor zone at the immune level. Altogether, the interface-derived T cells were different from those in normal and tumor zone.

### Macrophages show distinct phenotypes in interface zone

**Fig. 3:**
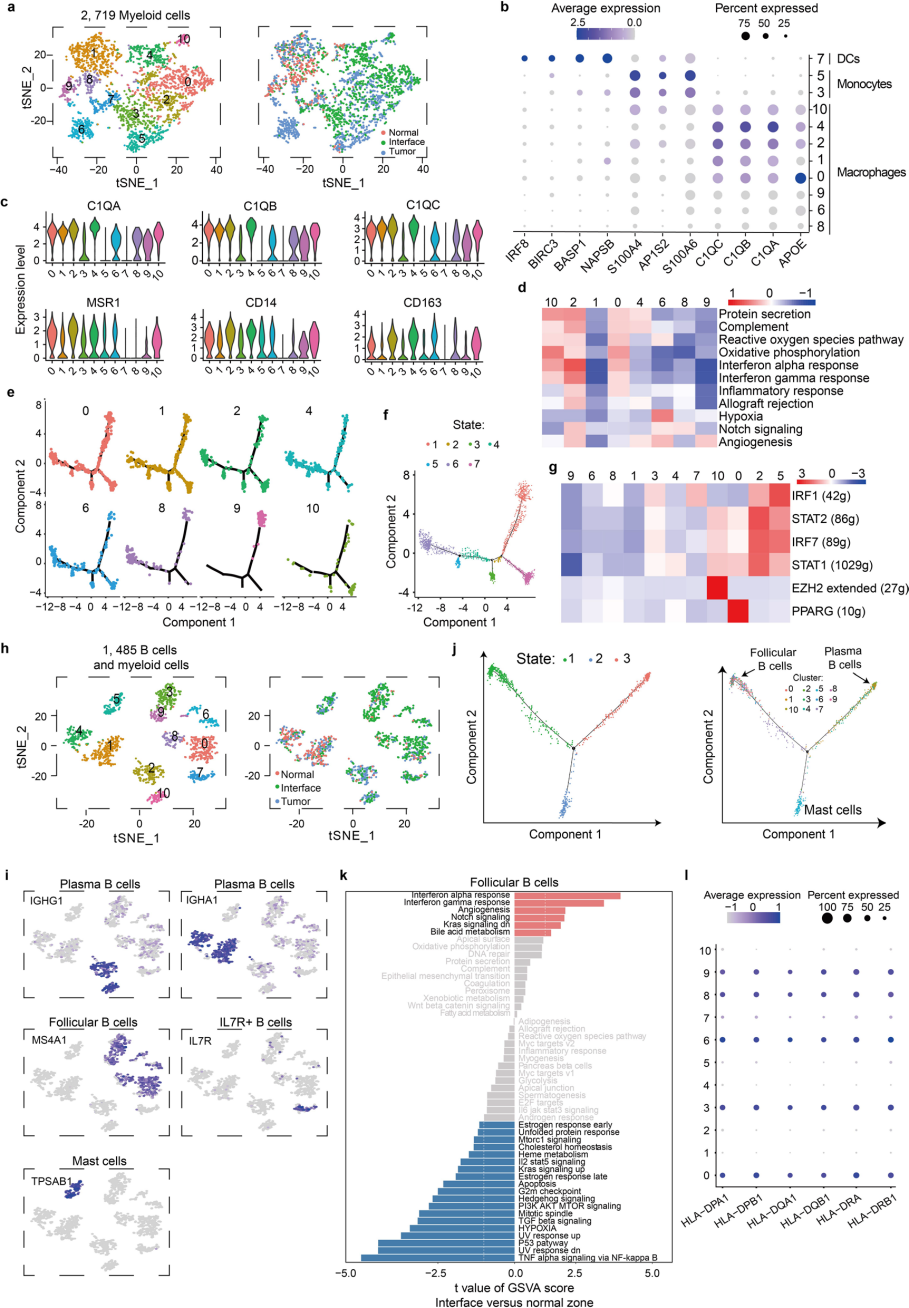
Myeloid cell and B cell clusters. **a** tSNE plots of 2,719 myeloid cells, color by relevant clusters (left) or sample type (right). **b** Bubble plots displaying expressions of specific genes in DCs (cluster 7), monocytes (clusters 3 and 5) and macrophages (clusters 0, 1, 2, 4, 6, 8, 9 and 10). **c** Violin plots displaying expressions of the selected marker genes in macrophage clusters. **d** Differences in pathway activity of each cell scored by GSVA. **e**–**f** Pseudotime trajectory of macrophage inferred by Monocle and showed by subcluster (e) and state (f). **g** Heatmap of single-cell regulon scores inferred by SCENIC. Number in brackets is the number of the target genes. g, genes. **h** tSNE plots of 1,485 B cells and myeloid cells, color by relevant clusters (left) or sample types (right). **i** Color coding for expression (gray to blue) of marker genes of plasma B cells, follicular B cells, IL7R + B cells and mast cells. **j** Pseudotime trajectory of B cell and mast cell state inferred by Monocle and characterized by state (left) and cell types (right). **k** Differences in pathway activity of each cell scored by GSVA. **l** Bubble plots displaying expressions of selected genes in subclusters

Tumor-associated myeloid cells can differentiate into different cellular subsets based on their microenvironment, and every subset has unique characteristics and plays different roles [27]. A total of 2719 myeloid cells were obtained and reclustered into 11 subclusters (Fig. 3a). Clusters 3 and 5, mainly enriched in the tumor and interface zones, were characterized by genes *S100A4, AP1S2 and S100A6* that defined monocytes [28, 29]. Cluster 7, across the three zones, was identified as dendritic cells (DCs) that selectively expressed DC markers *IRF8, BIRC3, BASP1* and *NAPSB* [30, 31]. The remaining clusters corresponded to macrophages that expressed *C1QA, C1QB, C1QC* and *APOE* enabling the distinction from monocytes (Fig. 3b) [32, 33]. In addition, *C1QA, C1QB* and *C1QC* genes could well distinguish macrophages from monocytes and DCs, and commonly used markers *MSR1*, *CD14* and *CD163* could not achieve this discrimination (Fig. 3c).

According to the analysis of pathway gene signatures, macrophage subtypes had different pathway activities (Fig. 3d). Decreased signals of inflammatory response, IFN response, reactive oxygen species and allograft rejection corresponded to M2-like macrophage phenotypes in cluster 9 that expressed scavenger receptors (SCARA3) (Fig. 3d, Additional file 1: Figure S2b), which indicated cluster 9, across the three zones, had phagocytic function in the body [34]. For tumor-derived cluster 6, a high expression level of *VEGFA* and a strong hypoxia signal supported that cluster 6 played an important role in promoting angiogenesis (Fig. 3d, Additional file 1: Figure S2b).

Clusters 0, 2 and 10 were mainly enriched in the tumor and interface zones and displayed a specificity of zone distribution of macrophages with strong IFN response and reactive oxygen species (Fig. 3d). Despite all this, the poor separation of these clusters indicated they did not represent separate entities but diverse cell states, which was consistent with a spectrum of macrophage activation states [35]. In order to determine the differentiation state of macrophages, we performed a pseudotime analysis by Monocle and found that macrophage subclusters did not have a specific polarization state, except for M2-like cluster 9 (Fig. 3e and f). Therefore, clusters 0, 2 and 10 did not belong to polarized macrophages. SCENIC analysis showed PPARG transcription factor as classical M2 marker was upregulated in cluster 0 (Fig. 3g) [36], indicating cluster 0 might tend to polarize to M2 macrophages. Transcription factors IRF7, IRF1, STAT2 and STAT1, which boosted inflammatory response, were upregulated in cluster 2, indicating cluster 2 might tend to polarize to M1 macrophages. Moreover, cluster 10 with high expression level of EZH2 had a function in tumor promotion [37]. Together, results displayed macrophages with various states in interface zone, and cluster 0 and cluster 10 may provide an environment for tumor occurrence in the interface zone.

### Follicular B cells and mast cells in interface and tumor zones possess specific characteristics

We detected 1,363 B cells and 122 mast cells that clustered near B cells. Given that TME could affect tumor infiltrating immune cells [16, 38], we explored the differences of B cells in three zones. Reclustering revealed 11 distinct subclusters (Fig. 3h), of which nine were enriched in tumor and interface zones and designated as plasma B cells (clusters 2 and 10, *IGHG1* +), follicular B cells (clusters 0, 3, 6, 8 and 9, *MS4A1* +), immature B cells (cluster 7, *IL7R* +) and mast cells (cluster 5, *TPSAB1* +) (Fig. 3i). The remaining two clusters were derived from all three zones and designated as plasma B cells with high level of *IGHA1* (clusters 1 and 4). Further, pseudotime analysis revealed the differentiation trajectory of B cells was divided into three branches corresponding to the main cell types follicular B cells, plasma B cells and mast cells (Fig. 3j). Wherein, immature B cells distributed along axes of the trajectory, indicating it was under a state of differentiation from follicular B cells to plasma B cells. Mast cells were independently located at one end of the differentiation trajectory because they did not belong to B cell lineage. Other clusters were well distributed at the corresponding location of cell types in the differentiation trajectory, indicating the differentiation process of follicular B cells to plasma B cells.

Pathway analysis showed no significant difference in plasma B cells among the three regions. However, interface-derived and tumor-derived follicular B cells expressed strong interferon response signal (Fig. 3k, Additional file 1: Figure S2c) and high levels of HLA class II molecules (Fig. 3l), which can enhance T cell-independent antibody response [39] and antigen presentation function. Additionally, interface-derived and tumor-derived mast cells had high expression levels of *VEGFA* and *VEGFB* genes (Additional file 1: Figure S2d), suggesting potential functions of promoting angiogenesis.

### Stem cells harbor different differentiation states in interface zone

**Fig. 4:**
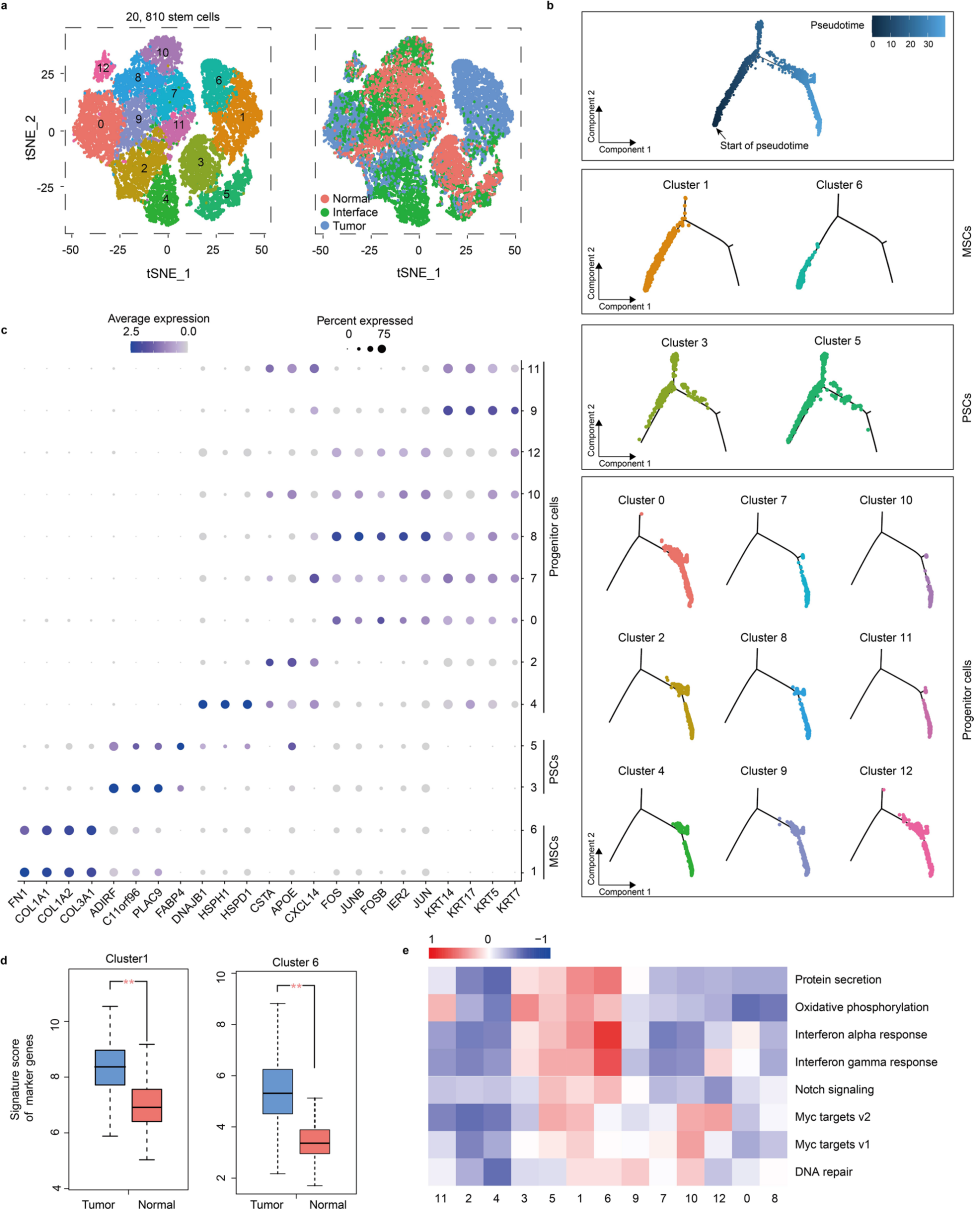
Stem cell clusters. **a** tSNE plots of 20,810 stem cells, color by relevant clusters (left) or sample type (right). **b** Pseudotime trajectory of stem cells state inferred by Monocle and characterized by subcluster (top to bottom): pseudotime characterized by a gradient from dark to light blue, pseudotime trajectory of MSCs, PSCs and progenitor cells. MSCs, Mesenchymal stem cells; PSCs, pluripotent stem cells. **c** Bubble plots displaying expressions of specific genes in MSCs (clusters 1 and 6), PSCs (clusters 3 and 5) and progenitor cells (clusters 0, 2, 4, 7, 8, 9, 10, 11 and 12). **d** Boxplots showing signature score of marker genes. The signature score is calculated by the average expression of log2(TPM + 1) of each gene in top 10 marker genes for MSCs (cluster 1 or 6) from breast cancer tissues (TCGA datasets, *n* = 1085) and normal breast tissues (TCGA and GTEx datasets, *n* = 291), **p < 0.01. **e** Heatmap of score differences of selected pathway activity of each cell by GSVA

A total of 20,810 stem cells were obtained and classified into 13 subclusters, among which clusters 1 and 6 were enriched in the interface and tumor zones, while cluster 4 was mainly derived from the interface zone, and the remaining clusters distributed in all three zones (Fig. 4a).

Pseudotime analysis yielded a connected trajectory with three main branches (Fig. 4b). Clusters 1 and 6 were markedly enriched at the start branch of pseudotime, suggesting they represented mesenchymal stem cells (*MSCs*, Fig. 4b), which was further confirmed by expression of *FN1*, a marker gene of MSCs (Fig. 4c) [40]. Besides, expressions of maker genes that separated clusters 1 and 6 from other clusters were confirmed in TCGA data (Fig. 4d). Interestingly, clusters 1 and 6 displayed high expressions of collagen type IA1, A2 and type IIIA1 (Fig. 4c), which have been previously reported to promote tumor growth and invasion. Clusters 3 and 5 were enriched at three zones that were merged into the first branch point (Fig. 4b), revealing the multilineage potential of these two clusters; thus, they were identified as pluripotent stem cells (PSCs). The remaining clusters were enriched at one end branch of the trajectory (Fig. 4b) and expressed some epithelial marker genes (Fig. 4c), so they were defined as epithelial progenitor cells.

Analysis of pathway gene signatures showed that each subtype had specific pathway activities (Fig. 4e). Wherein, MSCs (cluster1 and 6) had a strong IFN response signal, indicating the immunosuppressive capacity [41]. Notch signaling and myc targets had different expression levels between clusters 3 and 5, both of which belong to PSCs, suggesting PSCs possessed different differentiation and proliferation capabilities [42, 43]. Notably, interface-derived cluster 4 showed a weak DNA repair ability, which might promote breast carcinogenesis indicating a special interface microenvironment [44].

### Special endothelial cell subtypes in the interface and tumor zones

**Fig. 5:**
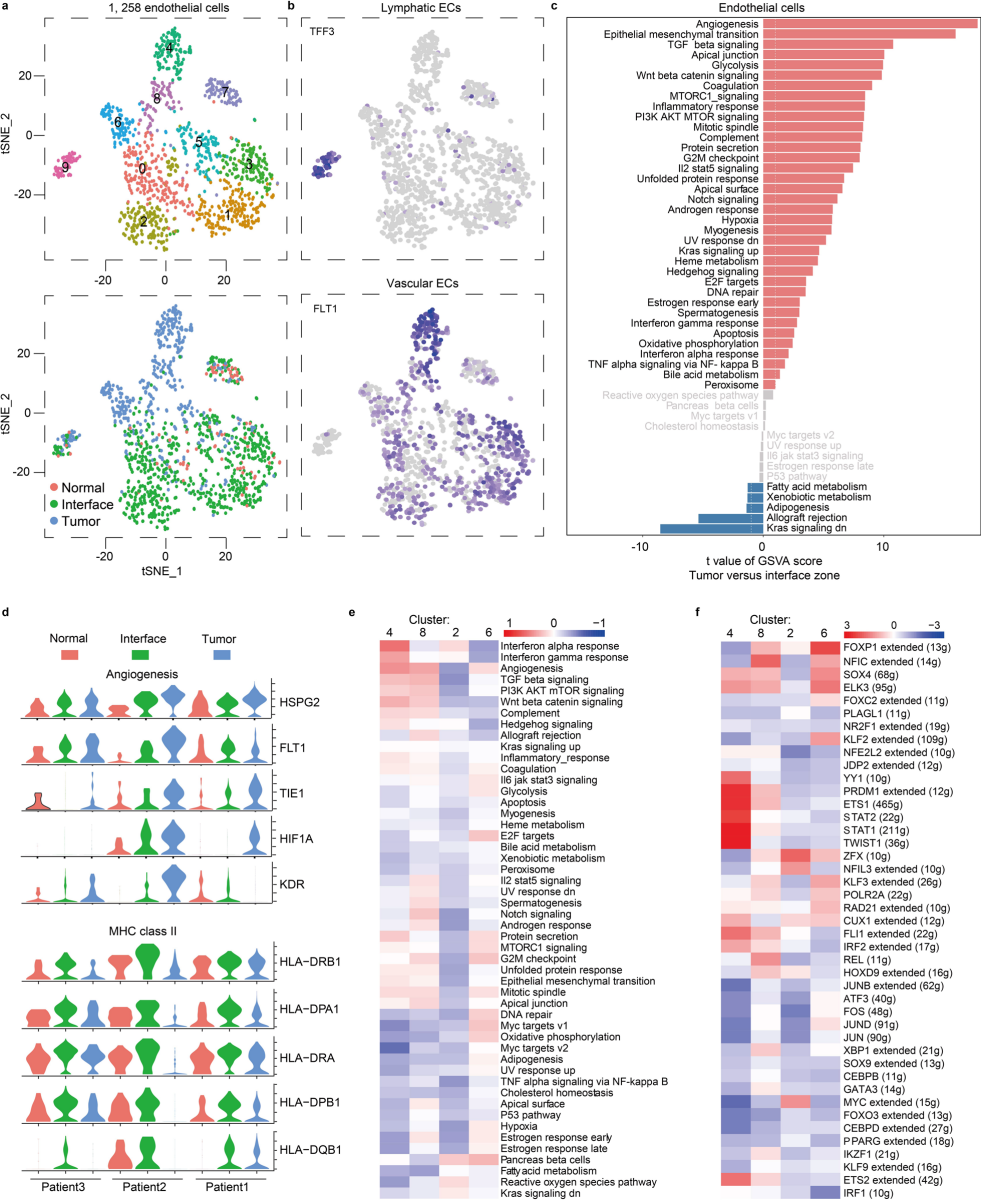
Endothelial cell clusters. **a** tSNE plots of 1258 endothelial cells, color by relevant clusters (top) or sample type (bottom). **b** Color coding for expressions (gray to blue) of marker genes of lymphatic and vascular endothelial cells. ECs, endothelial cells. **c** Differences in the score of pathway activity of each cell by GSVA, between endothelial cells isolated from tumor or interface zone. **d** Violin plots displaying expressions of the selected genes in endothelial cells from tumor, interface and normal zones. **e** Differences in pathway activity of each cell scored by GSVA between zone-specific vascular endothelial cells (clusters 2, 4, 6 and 8). **f** Heatmap of single-cell regulon scores inferred by SCENIC. Number in brackets is the number of the target genes. g, genes

Endothelial cells located between tissue and blood play a role in presenting tumor microenvironment signals. Here, we obtained 1258 endothelial cells and reclustered them into 10 clusters, and more interface-derived cells than normal-derived cells were observed among these clusters (Fig. 5a), indicating that endothelial cells in interface zone were strongly affected by tumor progression.

Based on marker genes for every cluster (Additional file 2: Table S5), we revealed one set of lymphatic endothelial cells (cluster 9, *TFF3* +) and nine sets of vascular endothelial cells (*FLT1* + , Fig. 5b): One was mostly interface-derived (clusters 2; *HLA-DQA2* +) and three were mostly tumor-derived (clusters 4, 6 and 8; *ROBO1* +) (Additional file 1: Figure S2e). The remaining clusters had no zone-specific enrichment. In tumor zone, we found that multiple pathway signals, especially related to angiogenesis, were enhanced compared with those in interface zone (Fig. 5c). Interestingly, angiogenesis-related molecules *HSPG2, FLT1, TIE1, HIF1A* and *KDR* gradually decreased from the tumor to the interface to the normal zone (Fig. 5d), suggesting that breast tissue in breast cancer patients should not be simply divided into tumor and normal zones, and that the interface zone should be considered. Additionally, interface-derived endothelial cells had increased expressions of MHC class II molecules *HLA-DRB1, DPA1, DRA, DPB1* and *DQB1* (Fig. 5d) and enhanced pathway activities of allograft rejection, hedgehog signaling and interferon response than those in normal zone (Additional file 1: Figure S2f), indicating that interface-derived endothelial cells were active in presenting antigen epitopes.

Given that clusters 2, 4, 6 and 8 were interface-specific or tumor-specific enrichment, we analyzed pathway gene signature and found a diversity of pathway activities among these clusters (Fig. 5e). Wherein, interface-derived cluster 2 had overall weak pathway signals. For tumor-derived clusters 4, 6 and 8, they also showed different pathway signal activities: Cluster 6 had weak interferon responses, but its G2M checkpoint and E2F target pathways were relatively strong compared with clusters 4 and 8, suggesting cells in cluster 6 were under a strong proliferative state.

Finally, we analyzed which transcription factors cause differences between tumor-derived clusters 4, 6, 8 and interface-derived cluster 2 using SCENIC (Fig. 5f). This identified KLF2 as a candidate transcription factor underlying the difference between cluster 6 and others, whereas genes regulated by ETS1 and ETS2 were responsible for specific cell phenotypes of clusters 4 and 8. Previous studies reported KLF2 promote cell proliferation [45] and ETS1 and ETS2 contribute to angiogenesis [46], indicating tumor-promoting functions of these tumor-derived clusters. Notably, interface-derived cluster2 expressed high levels of MYC transcription factor that was essential for vasculogenesis during tumor progression [47]. Therefore, we believed that some endothelial cells in both interface and tumor zones provided necessary conditions for the survival of tumor cells.

### Fibroblasts from interface zone share some characteristics with those from tumor zone

**Fig. 6:**
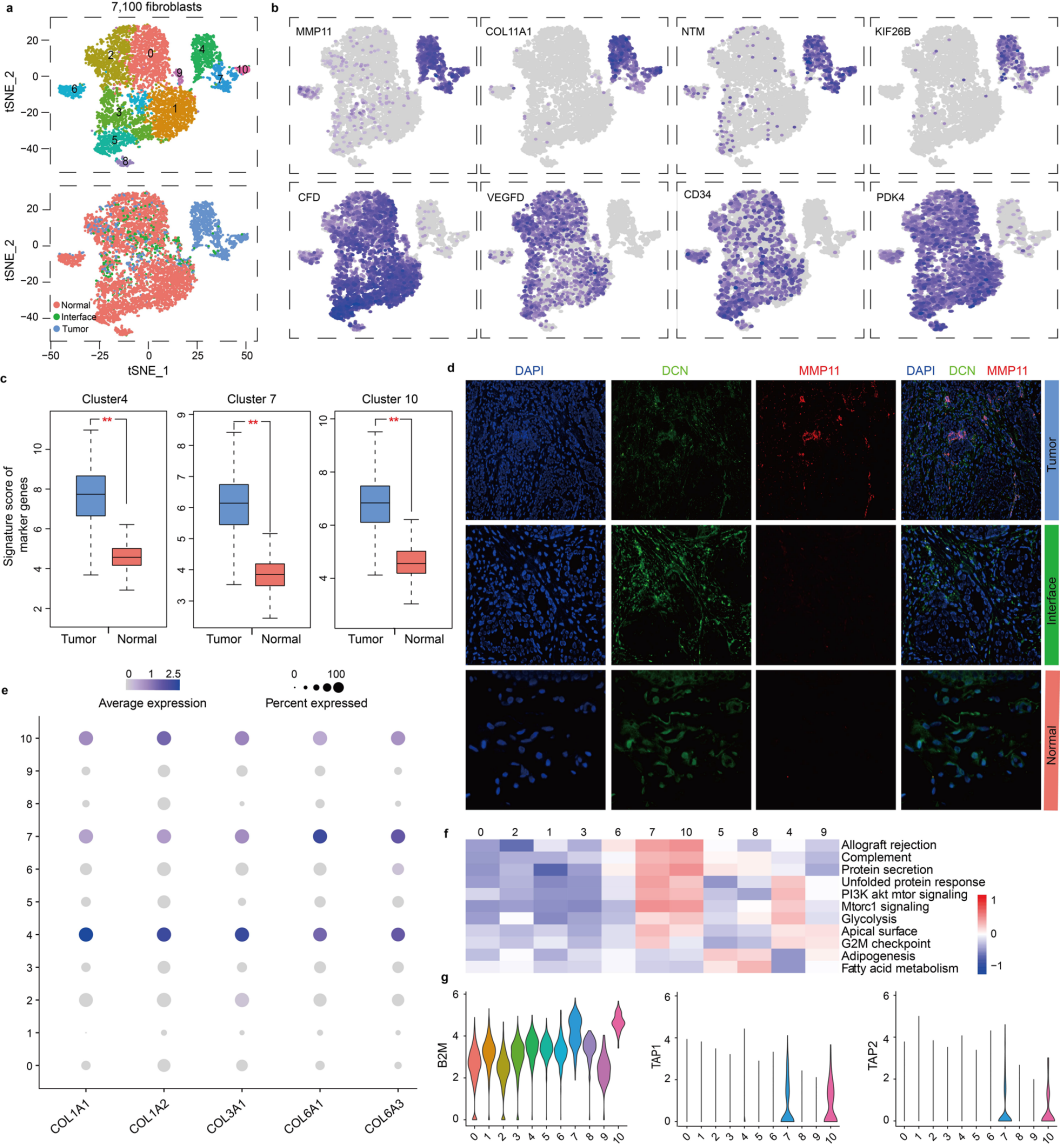
Fibroblast clusters. **a** tSNE plots of 7,100 fibroblasts, color by relevant clusters (top) or sample type (bottom). **b** Color coding for expression (gray to blue) of marker genes of fibroblasts. **c** Boxplots showing signature score of marker genes. The signature score is calculated by the average expression of log2(TPM + 1) of each gene in top 10 marker genes for cluster 4, 7 or 10 from breast cancer tissues (TCGA datasets, *n* = 1085) and normal breast tissues (TCGA and GTEx data, *n* = 291), **p < 0.01. **d** Target candidate staining of tumor, interface and normal zone. Nucleus stained by DAPI (blue), fibroblasts stained by DCN (green) and marker of interest depicted by MMP11 (red). DCN, decorin. MMP11, matrix metallopeptidase 11. Representative images were displayed. **e** Bubble plots displaying the expression of specific genes in fibroblast clusters. **f** Differences in pathway activity of each cell scored by GSVA between fibroblast clusters. **g** Violin plots displaying expressions of the selected genes in fibroblast clusters

Although the function of fibroblasts in wound healing has been well understood, their role in cancer still needs to be explored [48, 49]. Here, a total of 7100 fibroblasts were detected, and reclustering revealed 11 subclusters (Fig. 6a). No interface-specific cluster was observed, but clusters 4, 7 and 10 significantly enriched in the tumor zone. Clusters 4, 7 and 10 preferentially expressed *MMP11, COL11A1, NTM* and *KIF26B*, whereas other clusters showed high expressions of *CFD, VEGFD, CD34* and *PDK4* (Fig. 6b). Average expression levels of the top 10 marker genes of clusters 4, 7 and 10 were confirmed in tumor and normal breast tissues based on TCGA database (Fig. 6c). We further verified this result by detecting the expression of a representative marker gene *MMP11* in tissue sections through immunofluorescence staining (Fig. 6d). In additions, clusters 4, 7 and 10 expressed a variety of collagen proteins including COL1A1, COL1A2, COL3A1, COL6A1 and COL6A3 (Fig. 6e), which have been previously linked to abilities of proliferation, invasion, metastasis and drug resistance of tumor cells [50–53].

According to the analysis of pathway gene signatures, we observed a significant phenotypic diversity among all clusters (Fig. 6f). Clusters 4, 7 and 10 had an increased glycolysis, which may be responsible for their collagen synthesis [54]. PI3K/Akt/mTOR signaling, which has been reported to increase the migration of fibroblasts [55], was highly activated in clusters 4, 7 and 10. Besides, clusters 7 and 10 displayed high allograft rejection signature (Fig. 6f) and high expressions of MHC-related proteins B2M, TAP1 and TAP2 (Fig. 6g), indicating that they had roles of antigen presentation in TME and might induce expansion of regulatory T cells [56]. Although we did not find fibroblasts that only enriched in the interface zone, we observed a very small portion of interface-derived fibroblasts shared some characteristics with tumor zone-derived fibroblasts in clusters 4, 7 and 10 (Fig. 6a).

### Epithelial cells are heterogeneous and have distinctive subtypes in interface zone

**Fig. 7:**
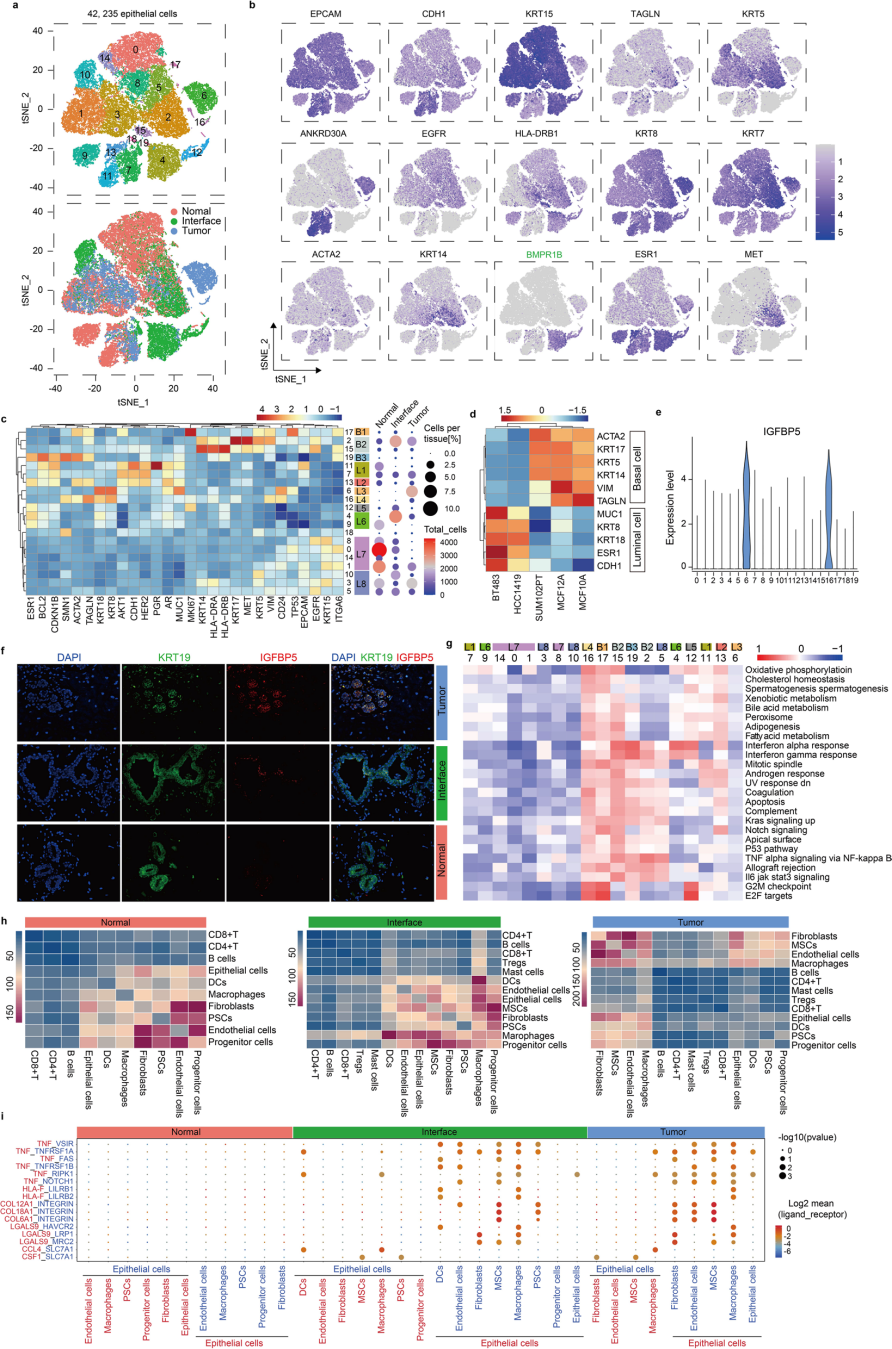
Epithelial cell clusters. **a** tSNE plots of 42,235 epithelial cells, color by relevant clusters (top) or sample type (bottom). **b** tSNE plots of the selected gene expressions of epithelial cell clusters from all samples. **c** Heatmap of the selected gene expressions for the 20 epithelial clusters (left) and percentage and total number of cells from tumor, interface and normal zones for per cluster (right). **d** Heatmap of expressions of epithelial lineage markers in cell lines. **e** Violin plot displaying expressions of the selected gene in epithelial cell clusters. **f** Target candidate staining of tumor, interface and normal zones. Nucleus stained by DAPI (blue), epithelial cells stained by KRT19 (green), and marker of interest depicted by IGFBP5 (red). IGFBP5, insulin-like growth factor binding protein 5. KRT19, keratin 19. **g** Differences in pathway activity of each cell scored by GSVA between epithelial cell clusters. **h** Heatmap showing the number of potential ligand–receptor pairs predicted by CellphoneDB between cell types in normal tissues, interface tissues and tumor zones, respectively. **i** Bubble plots showing ligand–receptor pairs of TNF, HLA-F and LGALS9 between epithelial cells and other cell types in normal, interface or tumor zones

Epithelial cells were the most abundant cells among all isolated cells from nine tissues. Twenty clusters were obtained by reclustering (Fig. 7a). The expression levels of epithelial markers revealed distinct cell phenotypes of these clusters (Fig. 7b). These clusters were further classified into eight luminal groups L1-L8 and three basal groups B1-B3 based on lineage marker gene expression patterns (Fig. 7c), and the reliability of these patterns was confirmed in breast epithelial cell lines by analyzing the data in a previous study (Fig. 7d) [57]. IGFBP5, a marker of tumor-derived clusters 6 and 16 (Fig. 7e), was further examined in tissue sections by immunofluorescence, and the result confirmed presence of these clusters as separate cellular entities (Fig. 7f). In particular, there are a total of 13,310 epithelial cells from the interface zone, of which 6,031 cells are from patient 1, 3,992 cells are from patient 2 and 3287 cells are from patient 3. Due to individual differences, the subtypes of these cells have patient preferences, for example, cluster 10 mainly derived from patient 3, clusters 4 and 12 mainly derived from patient 2, and cluster 2 derived from all three patients (Fig. 1d).

The heatmap showed the expression patterns of specific genes in epithelial subclusters (Fig. 7c). Group B1 was characterized by expressions of *KRT5, ACTA2* and *TAGLN* and displayed high levels of *MKI67* indicating a robust proliferative state. Group B2 was characterized by expressions of *KRT5, KRT14* and *KRT17* and showed a strong activity of immunogenicity and proliferation as indicated by receptor expressions of *HLA-DRA, HLA-DRB, MET* and *EGFR*. Wherein, about 54% of group B2 cells derived from interface zone.

Group B3 was characterized by expressions of *ACTA2* and *TAGLN*, and it only consisted of few cells with high expressions of proliferative markers *CDKN1B* and *BCL2*. In particular, the number of interface-derived cells was higher than that of tumor or normal-derived cells in group B1, B2 and B3, suggesting a unique property of the interface zone of breast tissues in breast cancer patients. Groups L1 and L2 were characterized by the expression of *CDH1*, a marker of luminal subtype. Wherein, cluster 11 was mature *HER2* + luminal B subtypes with a phenotype of *ESR1* + *PGR* + *HER2* + while cluster 7 was mature *HER2* + subtypes with a phenotype of *HER2* + *PGR-ESR1-*. Group L2 phenotypes showed high levels of *ESR1* and *HER2*, indicating *HER2* + luminal B subtypes. Meanwhile, group L2 expressed *ACTA2* and *TAGLN* representing an epithelial-to-mesenchymal transition (EMT) state. Groups L3 and L4 cells derived from the tumor zone and had low expressions of *ESR1/PGR/HER2*. Besides, group L4 presented EMT phenotypes as indicated by co-expressions of *KRT8/18, ACTA2* and *TAGLN* and proliferative phenotypes as indicated by *KI67* expression. About 89% of group L5 cells derived from the interface zone, and these cells showed high levels of *ESR1* and *MKI67*, representing a phenotype of luminal B cells. Group L6 showed a phenotype of luminal A cells with *ESR1* + *HER2-MKI67-* and expressed a low level of immunogenicity-related gene *HLA-DRB*. In group L6, cluster 4 was composed of 85% interface-derived cells while cluster 9 was composed of 99% normal-derived cells. Group L7 and L8 across the three zones co-expressed *EPCAM* and *ITGA6*, characteristics of progenitor cells [58].

In summary, luminal epithelial cells with low *ESR1/PGR/HER2* were mainly enriched in tumor zone, and they preferentially expressed some pro-proliferation genes and EMT markers, while proliferative luminal B cells were mainly enriched in the interface zone, suggesting that breast tissues of breast cancer patients should not be simply divided into tumor or normal zone, and that the interface zone with a special microenvironment should not be ignored.

We further characterized the features of these groups with distinct phenotypes by analysis of pathway gene signatures (Fig. 7g). Results showed that most signal pathways in the basal groups were active, but G2M check point, E2F targets, notch signaling and oxidative phosphorylation pathway were not consistent indicating basal cells across the three zones were heterogeneous. For tumor-derived groups L3 and L4, their differences were obvious. Group L4 was enriched for hallmarks of cell cycle (e.g., G2M checkpoint and E2F targets) compared with group L3, which was consistent with the gene expression patterns analyzed above (Fig. 7c). For groups L5 and L6, interface-derived group L5 had higher G2M checkpoint and E2F target activities than group L6, suggesting that luminal cells were distinct under the influence of the microenvironment in the interface zone.

Considering the interaction between epithelial cells and stromal cells in the surrounding microenvironment, we further analyzed the cell–cell interaction network between all types in the normal zone, interface zone and tumor zone based on expressions of ligand–receptor pairs, respectively (Fig. 7h). Epithelial cells in the interface zone showed interactions with most cell types, and they showed especially strong interactions with macrophages and MSCs.

Meanwhile, macrophages and MSCs also showed strong interactions with epithelial cells in the tumor zone, while there is no such interaction in the normal zone. Based on these findings, we concluded that cell–cell interaction in the interface zone was under a more active state and some characteristics of the interface zone is shared with the tumor zone. Undoubtedly, cells in the tumor zone had their specific properties, for instance, fibroblasts that can be named CAFs, had stronger interactions with epithelial cells compared with other stromal cells.

Besides, the bubble plots displayed that the ligand–receptor pairs between the epithelial cells and the stromal cells are stronger in the interface and the tumor zones than that in the normal zone including TNF, HLA, collagen and their receptor families (Fig. 7i). Epithelial cells had a much greater effect on stromal cells than stromal cells have on epithelial cells in both the interface and tumor zones, which implied that epithelial cells play crucial roles in the formation of tumor or para-carcinoma microenvironment. For instance, interface- and tumor-derived epithelial cells expressed TNF, and endothelial cells expressed notch1, which was able to protect vascular endothelial cells from TNF-induced apoptosis [59], but endothelial cells almost had no effect on epithelial cells based on expressions of interaction pairs.

### Functions of a selected gene BMPR1B differentially expressed in epithelial cells among three zones

**Fig. 8:**
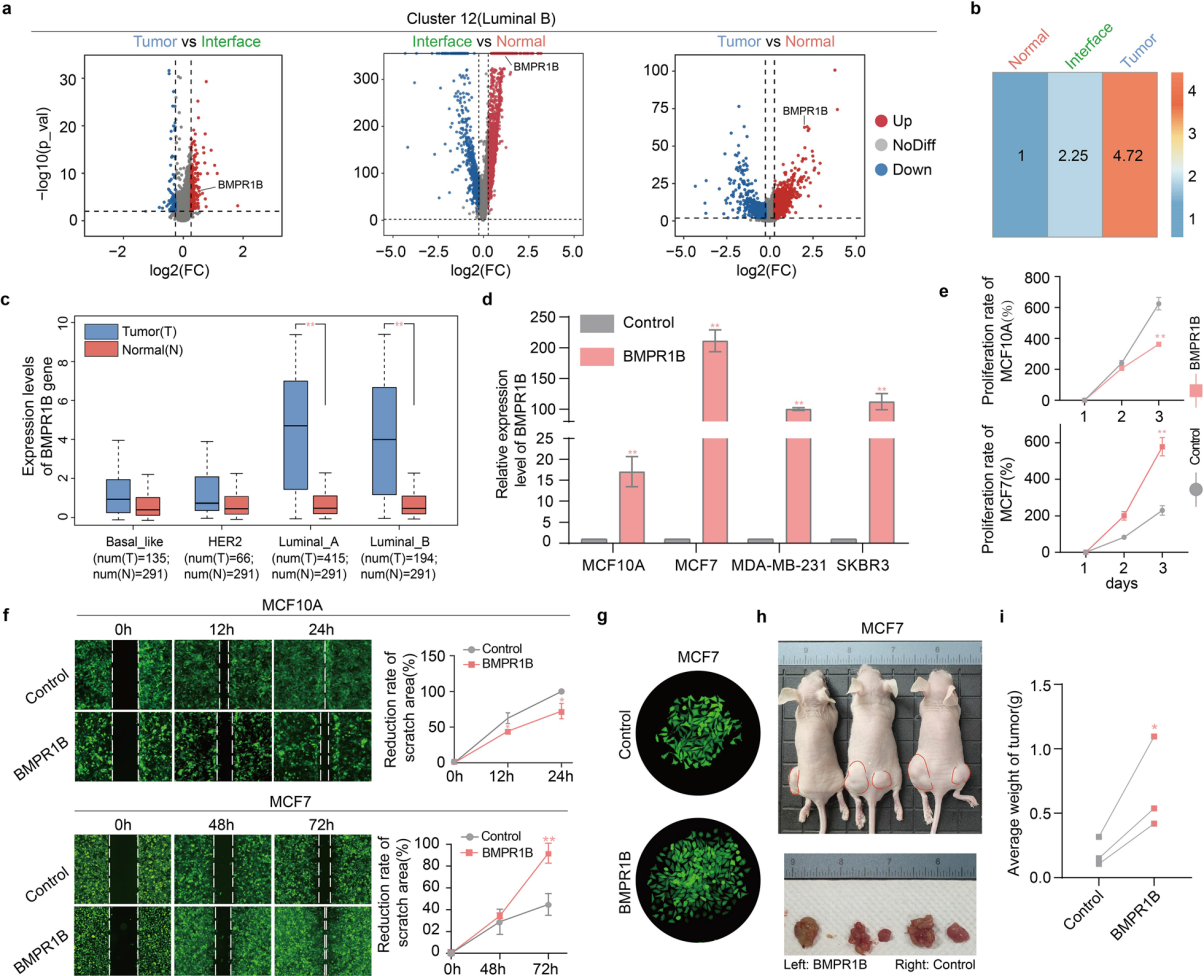
Expression and function of BMPR1B gene in epithelial cells. **a** Volcano plots of BMPR1B gene expression in cluster 12 from different zones here (left to right): Tumor zone versus interface zone, interface zone versus normal zone and tumor zone versus normal zone. **b** Heatmap of the average expression trend of BMPR1B gene in luminal cells across the three zones (normal, interface and tumor). **c** Box plots showing the log2(TPM + 1) value of BMPR1B gene in basal-like breast cancer (TCGA datasets, *n* = 135), HER2 + breast cancer (TCGA datasets, *n* = 66), luminal A breast cancer (TCGA datasets, *n* = 415), luminal B breast cancer (TCGA datasets, *n* = 194) and normal breast tissues (TCGA and GTEx data, *n* = 291), **p < 0.01. **d** BMPR1B overexpression in different types of epithelial cells was determined by RT-qPCR analysis. Matched cell lines with raw vector were used as control group, **p < 0.01 vs. control. **e** Line chart of cell proliferation measured at 450 nm, **p < 0.01. **f** Wound-healing assay of MCF10A and MCF-7 cells with or without BMPR1B overexpression. Left: Representative images of BMPR1B-overexpressed cells and control at three time point after wounding. Right: quantification of the reduction rate of scratch area (mean ± SD). **g** Photographs of cell clones observed under a microscope. BMPR1B gene promoted the colony forming ability of MCF7 cells. **h** Photographs of BALB/cNj-Foxn1^nu^/Gpt mice. BMPR1B-overexpressed MCF-7 cells and control cells were injected into subcutaneous location of left and right groin in nude mice, respectively. 15th day post-transplantation, mice were photographed, and tumors were removed surgically and weighed. **i** Statistical analysis of mice tumor weights (*n* = 3, *p < 0.05, *t* test)

We performed an in-depth study on a specific gene of *BMPR1B* that was highly expressed in cluster 4 (luminal A) and 12 (luminal B), which were mainly enriched in the interface zone (Fig. 7a and b). By analyzing the differential genes in cluster 12, we further found that the average expression level of *BMPR1B* gene in tumor-derived epithelial cells was higher than that in interface-derived epithelial cells (Fig. 8a, left). In addition, the average expression level of *BMPR1B* was higher in both the interface- and tumor-derived epithelial cells in cluster 12 than in all luminal epithelial cells in the normal zone (Fig. 8a, middle and right). *BMPR1B* had the same expression trend in cluster 4 as in cluster 12 (Additional file 1: Figure S3a). As a whole, the average expression level of *BMPR1B* gradually increased from the normal to the interface to the tumor-derived luminal cells (Fig. 8b). The expression of *BMPR1B* gene between tumor and normal zones is consistent with the TCGA database (Fig. 8c).

To explore functions of *BMPR1B* gene, we first overexpressed *BMPR1B* gene in non-tumorigenic MCF10A breast cell line (Fig. 8d) and found that *BMPR1B* overexpression inhibited the proliferation (Fig. 8e, up) and migration (Fig. 8f, up) of MCF10A cells. However, tumor-bearing experiment showed *BMPR1B* did not change the non-tumorigenic property of MCF10A cells in vivo.

Next, we investigated functions of *BMPR1B* gene in breast cancer cells with different phenotypes including MCF-7 (luminal type), SKBR3 (HER2 + type) and MDA-MB-231 (triple-negative breast cancer) by stably overexpressing *BMPR1B* gene (Fig. 8d). For MCF-7 cells, *BMPR1B* overexpression could enhance proliferation (Fig. 8e, down), migration (Fig. 8f, down) and clone formation (Fig. 8g) of the MCF7 cells in vitro and could promote tumor formation and growth in vivo (Fig. 8h and i). For MDA-MB-231 cells, *BMPR1B* overexpression promoted cell proliferation (Additional file 1: Figure S3b) and clone formation (Additional file 1: Figure S3c), but it did not affect cell migration (Additional file 1: Figure S3d) and tumorigenicity in vivo (Additional file 1: Figure S3e). For SKBR3 cells, BMPR1B overexpression did not change proliferation (Additional file 1: Figure S3b), clone formation (Additional file 1: Figure S3c) or migration of the SKBR3 cells (Additional file 1: Figure S3d). These findings suggest that *BMPR1B* gene may play crucial roles in the development of luminal breast carcinoma.

## Discussion

Here we characterized a landscape of breast cancer tissue at single-cell resolution based on the concept of the existing three zones (i.e., tumor zone, normal zone and interface zone) in the breast tissue of breast cancer patients [13]. This landscape was a comprehensive exploration of breast TME where the interface zone is considered a unique functional and molecular zone between the tumor invasion front and the normal tissue zone. Although not all subtypes were fully described, we confirmed some key differences in each zone. By analyzing differences of major cell types in each zone, we confirmed a unique microenvironment of the interface zone. For instance, some specific epithelial cell subtypes only existed in the interface zone. This could provide a new reference for the study of tumor development and invasion and the determination of surgical excision boundary.

We found ten major stromal cell subtypes in three zones of breast tissue of breast cancer patients: endothelial cells, fibroblasts, macrophages, monocytes, DCs, MSCs, PSCs, progenitor cells, and T and B lymphoid cells. We described key phenotypes about these cell types. In terms of immune cells, we found that more regulatory T cells were distributed in the interface zone than in the normal zone, and they expressed strong angiogenesis-related molecules and high level of immune checkpoint molecules, which are conducive to the survival of tumor cells. Follicular B cells were abundant in the interface zone and exhibited T cell-independent antibody response. However, their functions across breast tissues are not significantly different after follicular B cells differentiate into plasma cells. Surprisingly, mast cells had functions of promoting angiogenesis in the interface zone. Macrophages were susceptible to the microenvironment and exhibited a variety of states in the interface zone. For instance, cluster 10 showed a tumor-promoting role as indicated by the high expression of EZH2, and cluster 0 had high expression level of the transcription factor PPARG that can regulate the production of M2-like macrophages. The expression pattern of these immune cells in the interface zone were similar but different from that in the tumor zone. This shows that immune cells of the interface zone can provide conditions for the occurrence or the development of the tumor, but they interestingly do not resemble the immune cells of the tumor zone.

Besides immune cells, we also analyzed other stromal cell types in breast tissues. For endothelial cells, expressions of angiogenesis-related genes gradually decreased from the tumor to the interface to the normal zone. In addition, expression levels of antigen presentation-related genes of the interface-derived endothelial cells were higher than those derived from the tumor and the normal zones, which indicated that the interface-derived endothelial cells actively participate in signal presentation between blood and tissues. Therefore, endothelial cells in neither the tumor nor the normal zone can represent those in the interface zone.

For fibroblasts, some clusters (clusters 4, 7 and 10) were mainly enriched in the tumor zone with a small portion of the interface-derived fibroblasts. These clusters represented cancer-associated fibroblasts (CAFs) based on the definition of CAFs that are stated as fibroblasts within or adjacent to a tumor [60]. This implied that some fibroblasts in the interface zone had been affected by the neighboring tumor because some interface-derived fibroblasts in clusters 4, 7 and 10 shared common characteristics with the tumor-derived fibroblasts. In addition, clusters 4, 7 and 10 were separated based on their gene expression profiles indicating a heterogenicity of CAFs.

Among all stem cell subpopulations, MSCs were mainly enriched in the interface and tumor zones and expressed collagens that could promote the growth and invasion of tumor cells. Notably, interface-derived epithelial progenitor cells (cluster 4) had the weakest DNA repair activities among all progenitor cells, which can easily lead to gene mutation [61], thus inducing cancer cell formation. This might provide a possibility that not all tumor infiltration processes are caused by the outward invasion of cancer cells from the tumor zone, but the continuous emergence of new cancer cells in the interface zone which may contribute to the formation of tumor infiltration.

For epithelial cells, they were grossly divided into two types luminal and basal populations. Group L7 and L8 that were progenitor cells based on their enrichment in the interface zone genes were clustered into the tSNE plot of epithelial cells. In each zone, there was a distribution of cells with specific phenotypes. For instance, luminal cells with low *ESR1/PGR/HER2* were enriched in the tumor zone, and luminal B cells (L5) were enriched in the interface zone. These findings suggested that the interface zone has a special microenvironment that is different from the normal or tumor zones. We investigated the functions of a gene BMPR1B whose expression gradually increased in luminal cells from the normal to the interface to the tumor zone. We found that *BMPR1B* overexpression could enhance the malignant biological behaviors of MCF-7 breast cancer cells in both in vitro and in vivo experiments, suggesting that the interface zone possesses some characteristics like the tumor zone. In addition, we found that the proliferative luminal cells (KI67 +) in the interface zone of HER2 positive breast tumors highly expressed the BMPR1B gene and may develop into cancer cells in this specific microenvironment. This may suggest that it would be beneficial to remove the interface tissue zone during breast conservation surgery given the presence of proliferative luminal cells in HER2 positive breast tumors.

## Conclusions

In summary, we described cellular and molecular profiles of human breast tissues based on a taxonomic concept of the existence of three zones in human breast tissue (a normal zone, an interface zone and a tumor zone) through single-cell RNA-seq analysis. This suggests interface zone could be easily affected by TME remodeling and possess unique characteristics. This single-cell landscape serves as a basis for studying the occurrence and invasion of breast cancer.

### Supplementary Information

**Additional file 1**. **Table S1** Clinical parameters of the three breast cancer patients. **Table S2** PCR primer sequence. **Table S3** The specific information for each sample. **Table S4** Numbers of immune cells with immune-hot and immune-cold activities. **Fig. S1** Identification and collection of representative samples. **Fig. S2** Gene expression and pathway analysis in stromal cells. **Fig. S3** Expression and function of BMPR1B gene in epithelial cells.

**Additional file 2**. **Table S5** Specific information for marker genes.

## Data Availability

The single-cell RNA sequencing data and bulk-seq data are available in Sequence Read Archive (Access number: SRP434502).

## Abbreviations

BCS: Breast conserving surgery
TME: Tumor microenvironment
scRNA-seq: Single-cell RNA sequencing
GEMs: Gel bead-in emulsions
TF: Transcription factor
RT-qPCR: Real-time quantitative polymerase chain reaction
TCGA: The Cancer Genome Atlas
NK cells: Natural killer cells
SCENIC: Single-cell regulatory network inference and clustering
DCs: Dendritic cells
MSCs: Mesenchymal stem cells
PSCs: Pluripotent stem cells
EMT: Epithelial-to-mesenchymal transition
CAFs: Cancer-associated fibroblasts

## References

[1] Sung H, Ferlay J, Siegel RL, Laversanne M, Soerjomataram I, Jemal A, Bray F (2021). Global cancer statistics 2020: GLOBOCAN estimates of incidence and mortality worldwide for 36 cancers in 185 countries. CA Cancer J Clin.

[2] Harbeck N, Gnant M (2017). Breast cancer. Lancet.

[3] Maughan KL, Lutterbie MA, Ham PS (2010). Treatment of breast cancer. Am Fam Physician.

[4] Pilewskie M, Morrow M (2018). Margins in breast cancer: How much is enough?. Cancer.

[5] Kim BG, An HJ, Kang S, Choi YP, Gao MQ, Park H, Cho NH (2011). Laminin-332-rich tumor microenvironment for tumor invasion in the interface zone of breast cancer. Am J Pathol.

[6] Balasundaram G, Krafft C, Zhang R, Dev K, Bi R, Moothanchery M, Popp J, Olivo M (2021). Biophotonic technologies for assessment of breast tumor surgical margins-a review. J Biophotonics.

[7] Xiao Y, Yu D (2021). Tumor microenvironment as a therapeutic target in cancer. Pharmacol Ther.

[8] Bartoschek M, Oskolkov N, Bocci M, Lovrot J, Larsson C, Sommarin M, Madsen CD, Lindgren D, Pekar G, Karlsson G (2018). Spatially and functionally distinct subclasses of breast cancer-associated fibroblasts revealed by single cell RNA sequencing. Nat Commun.

[9] Azizi E, Carr AJ, Plitas G, Cornish AE, Konopacki C, Prabhakaran S, Nainys J, Wu K, Kiseliovas V, Setty M (2018). Single-cell map of diverse immune phenotypes in the breast tumor microenvironment. Cell.

[10] Quinting T, Heymann AK, Bicker A, Nauth T, Bernardini A, Hankeln T, Fandrey J, Schreiber T (2021). Myoglobin protects breast cancer cells due to Its ROS and NO scavenging properties. Front Endocrinol.

[11] Quail DF, Joyce JA (2013). Microenvironmental regulation of tumor progression and metastasis. Nat Med.

[12] Alizadeh AA, Aranda V, Bardelli A, Blanpain C, Bock C, Borowski C, Caldas C, Califano A, Doherty M, Elsner M (2015). Toward understanding and exploiting tumor heterogeneity. Nat Med.

[13] Gao MQ, Kim BG, Kang S, Choi YP, Park H, Kang KS, Cho NH (2010). Stromal fibroblasts from the interface zone of human breast carcinomas induce an epithelial-mesenchymal transition-like state in breast cancer cells in vitro. J Cell Sci.

[14] Ahmed R, Zaman T, Chowdhury F, Mraiche F, Tariq M, Ahmad IS, Hasan A (2022). Single-cell RNA sequencing with spatial transcriptomics of cancer tissues. Int J Mol Sci.

[15] Lambrechts D, Wauters E, Boeckx B, Aibar S, Nittner D, Burton O, Bassez A, Decaluwe H, Pircher A, Van den Eynde K (2018). Phenotype molding of stromal cells in the lung tumor microenvironment. Nat Med.

[16] Chung W, Eum HH, Lee HO, Lee KM, Lee HB, Kim KT, Ryu HS, Kim S, Lee JE, Park YH (2017). Single-cell RNA-seq enables comprehensive tumour and immune cell profiling in primary breast cancer. Nat Commun.

[17] Davis RT, Blake K, Ma D, Gabra MBI, Hernandez GA, Phung AT, Yang Y, Maurer D, Lefebvre A, Alshetaiwi H (2020). Transcriptional diversity and bioenergetic shift in human breast cancer metastasis revealed by single-cell RNA sequencing. Nat Cell Biol.

[18] Kang S, Kim MJ, An H, Kim BG, Choi YP, Kang KS, Gao MQ, Park H, Na HJ, Kim HK (2010). Proteomic molecular portrait of interface zone in breast cancer. J Proteome Res.

[19] Shan LH, Sun WG, Han W, Qi L, Yang C, Chai CC, Yao K, Zhou QF, Wu HM, Wang LF (2012). Roles of fibroblasts from the interface zone in invasion, migration, proliferation and apoptosis of gastric adenocarcinoma. J Clin Pathol.

[20] Wada J, Suzuki H, Fuchino R, Yamasaki A, Nagai S, Yanai K, Koga K, Nakamura M, Tanaka M, Morisaki T (2009). The contribution of vascular endothelial growth factor to the induction of regulatory T-cells in malignant effusions. Anticancer Res.

[21] Tanabe Y, Nishibori T, Su L, Arduini RM, Baker DP, David M (2005). Cutting edge: role of STAT1, STAT3, and STAT5 in IFN-alpha beta responses in T lymphocytes. J Immunol.

[22] Nguyen KB, Cousens LP, Doughty LA, Pien GC, Durbin JE, Biron CA (2000). Interferon alpha/beta-mediated inhibition and promotion of interferon gamma: STAT1 resolves a paradox. Nat Immunol.

[23] Honda K, Taniguchi T (2006). IRFs: master regulators of signalling by Toll-like receptors and cytosolic pattern-recognition receptors. Nat Rev Immunol.

[24] Itahashi K, Irie T, Yuda J, Kumagai S, Tanegashima T, Lin YT, Watanabe S, Goto Y, Suzuki J, Aokage K (2022). BATF epigenetically and transcriptionally controls the activation program of regulatory T cells in human tumors. Sci Immunol.

[25] Grunwald BT, Devisme A, Andrieux G, Vyas F, Aliar K, McCloskey CW, Macklin A, Jang GH, Denroche R, Romero JM (2021). Spatially confined sub-tumor microenvironments in pancreatic cancer. Cell.

[26] Galon J, Bruni D (2019). Approaches to treat immune hot, altered and cold tumours with combination immunotherapies. Nat Rev Drug Discov.

[27] Cha YJ, Koo JS (2020). Role of tumor-associated myeloid cells in breast cancer. Cells.

[28] Missarova A, Jain J, Butler A, Ghazanfar S, Stuart T, Brusko M, Wasserfall C, Nick H, Brusko T, Atkinson M (2021). geneBasis: an iterative approach for unsupervised selection of targeted gene panels from scRNA-seq. Genome Biol.

[29] Young MD, Mitchell TJ, Vieira Braga FA, Tran MGB, Stewart BJ, Ferdinand JR, Collord G, Botting RA, Popescu DM, Loudon KW (2018). Single-cell transcriptomes from human kidneys reveal the cellular identity of renal tumors. Science.

[30] Wang Z, Li Z, Zhou K, Wang C, Jiang L, Zhang L, Yang Y, Luo W, Qiao W, Wang G (2021). Deciphering cell lineage specification of human lung adenocarcinoma with single-cell RNA sequencing. Nat Commun.

[31] Balzer MS, Rohacs T, Susztak K (2022). How many cell types are in the kidney and what do they do?. Annu Rev Physiol.

[32] Pombo Antunes AR, Scheyltjens I, Lodi F, Messiaen J, Antoranz A, Duerinck J, Kancheva D, Martens L, De Vlaminck K, Van Hove H (2021). Single-cell profiling of myeloid cells in glioblastoma across species and disease stage reveals macrophage competition and specialization. Nat Neurosci.

[33] Ge G, Han Y, Zhang J, Li X, Liu X, Gong Y, Lei Z, Wang J, Zhu W, Xu Y (2022). Single-cell RNA-seq reveals a developmental hierarchy super-imposed over subclonal evolution in the cellular ecosystem of prostate cancer. Adv Sci.

[34] Shapouri-Moghaddam A, Mohammadian S, Vazini H, Taghadosi M, Esmaeili SA, Mardani F, Seifi B, Mohammadi A, Afshari JT, Sahebkar A (2018). Macrophage plasticity, polarization, and function in health and disease. J Cell Physiol.

[35] Xue J, Schmidt SV, Sander J, Draffehn A, Krebs W, Quester I, De Nardo D, Gohel TD, Emde M, Schmidleithner L (2014). Transcriptome-based network analysis reveals a spectrum model of human macrophage activation. Immunity.

[36] Mukhopadhyay D, Mukherjee S, Roy S, Dalton JE, Kundu S, Sarkar A, Das NK, Kaye PM, Chatterjee M (2015). M2 polarization of monocytes-macrophages is a hallmark of Indian Post Kala-Azar Dermal Leishmaniasis. PLoS Negl Trop Dis.

[37] Chen X, Chen Y, Chen X, Wei P, Lin Y, Wu Z, Lin Z, Kang D, Ding C (2022). Single-cell RNA sequencing reveals intra-tumoral heterogeneity of glioblastoma and a pro-tumor subset of tumor-associated macrophages characterized by EZH2 overexpression. Biochim Biophys Acta Mol Basis Dis.

[38] Anastasiou D (2017). Tumour microenvironment factors shaping the cancer metabolism landscape. Br J Cancer.

[39] Swanson CL, Wilson TJ, Strauch P, Colonna M, Pelanda R, Torres RM (2010). Type I IFN enhances follicular B cell contribution to the T cell-independent antibody response. J Exp Med.

[40] Benvenuto M, Focaccetti C, Izzi V, Masuelli L, Modesti A, Bei R (2021). Tumor antigens heterogeneity and immune response-targeting neoantigens in breast cancer. Semin Cancer Biol.

[41] Kwee BJ, Lam J, Akue A, KuKuruga MA, Zhang K, Gu L, Sung KE (2021). Functional heterogeneity of IFN-gamma-licensed mesenchymal stromal cell immunosuppressive capacity on biomaterials. Proc Natl Acad Sci USA.

[42] Makino K, Long MD, Kajihara R, Matsueda S, Oba T, Kanehira K, Liu S, Ito F (2022). Generation of cDC-like cells from human induced pluripotent stem cells via Notch signaling. J Immunother Cancer.

[43] Qi H, Pei D (2007). The magic of four: induction of pluripotent stem cells from somatic cells by Oct4, Sox2, Myc and Klf4. Cell Res.

[44] Stead ER, Bjedov I (2021). Balancing DNA repair to prevent ageing and cancer. Exp Cell Res.

[45] Ling L, Wang HF, Li J, Li Y, Gu CD (2020). Downregulated microRNA-92a-3p inhibits apoptosis and promotes proliferation of pancreatic acinar cells in acute pancreatitis by enhancing KLF2 expression. J Cell Biochem.

[46] Wei G, Srinivasan R, Cantemir-Stone CZ, Sharma SM, Santhanam R, Weinstein M, Muthusamy N, Man AK, Oshima RG, Leone G (2009). Ets1 and Ets2 are required for endothelial cell survival during embryonic angiogenesis. Blood.

[47] Baudino TA, McKay C, Pendeville-Samain H, Nilsson JA, Maclean KH, White EL, Davis AC, Ihle JN, Cleveland JL (2002). c-Myc is essential for vasculogenesis and angiogenesis during development and tumor progression. Genes Dev.

[48] Gabbiani G, Ryan GB, Majne G (1971). Presence of modified fibroblasts in granulation tissue and their possible role in wound contraction. Experientia.

[49] Ohlund D, Elyada E, Tuveson D (2014). Fibroblast heterogeneity in the cancer wound. J Exp Med.

[50] Menke A, Philippi C, Vogelmann R, Seidel B, Lutz MP, Adler G, Wedlich D (2001). Down-regulation of E-cadherin gene expression by collagen type I and type III in pancreatic cancer cell lines. Cancer Res.

[51] Chintala SK, Sawaya R, Gokaslan ZL, Rao JS (1996). The effect of type III collagen on migration and invasion of human glioblastoma cell lines in vitro. Cancer Lett.

[52] Chen P, Cescon M, Bonaldo P (2013). Collagen VI in cancer and its biological mechanisms. Trends Mol Med.

[53] Sherman-Baust CA, Weeraratna AT, Rangel LB, Pizer ES, Cho KR, Schwartz DR, Shock T, Morin PJ (2003). Remodeling of the extracellular matrix through overexpression of collagen VI contributes to cisplatin resistance in ovarian cancer cells. Cancer Cell.

[54] Nigdelioglu R, Hamanaka RB, Meliton AY, O’Leary E, Witt LJ, Cho T, Sun K, Bonham C, Wu D, Woods PS (2016). Transforming growth factor (TGF)-beta promotes de novo serine synthesis for collagen production. J Biol Chem.

[55] Xiao W, Tang H, Wu M, Liao Y, Li K, Li L, Xu X (2017). Biosci Rep.

[56] Huang H, Wang Z, Zhang Y, Pradhan RN, Ganguly D, Chandra R, Murimwa G, Wright S, Gu X, Maddipati R (2022). Mesothelial cell-derived antigen-presenting cancer-associated fibroblasts induce expansion of regulatory T cells in pancreatic cancer. Cancer Cell.

[57] Heiser LM, Sadanandam A, Kuo WL, Benz SC, Goldstein TC, Ng S, Gibb WJ, Wang NJ, Ziyad S, Tong F (2012). Subtype and pathway specific responses to anticancer compounds in breast cancer. Proc Natl Acad Sci USA.

[58] Stingl J, Eaves CJ, Zandieh I, Emerman JT (2001). Characterization of bipotent mammary epithelial progenitor cells in normal adult human breast tissue. Breast Cancer Res Treat.

[59] Fortini F, Vieceli Dalla Sega F, Caliceti C, Aquila G, Pannella M, Pannuti A, Miele L, Ferrari R, Rizzo P (2017). Estrogen receptor beta-dependent Notch1 activation protects vascular endothelium against tumor necrosis factor alpha (TNFalpha)-induced apoptosis. J Biol Chem.

[60] Chen Y, McAndrews KM, Kalluri R (2021). Clinical and therapeutic relevance of cancer-associated fibroblasts. Nat Rev Clin Oncol.

[61] Hopkins JL, Lan L, Zou L (2022). DNA repair defects in cancer and therapeutic opportunities. Genes Dev.

