# Supplementary material for: Single-cell RNA reveals a tumorigenic microenvironment in the interface zone of human breast tumors
Source: Breast Cancer Res. 2023 Aug 29;25:100. doi: 10.1186/s13058-023-01703-7 (PMC10463980; doi:10.1186/s13058-023-01703-7)
Supplement: Supplementary file 1 — Additional file 1. Table S1 Clinical parameters of the three breast cancer patients. Table S2 PCR primer sequence. Table S3 The specific information for each sample. Table S4 Numbers of immune cells with immune-hot and immune-cold activities. Fig. S1 Identification and collection of representative samples. Fig. S2 Gene expression and pathway analysis in stromal cells. Fig. S3 Expression and function of BMPR1B gene in epithelial cells. [file 13058_2023_1703_MOESM1_ESM.pdf]

Single-cell RNA reveals a tumorigenic microenvironment in the interface zone of human breast tumors

Wei Yang[1], Meiyu Xu[1], Shuoqi Xu[1], Qingxian Guan[1], Shuaiming Geng[1], Juanhong Wang[2], Wei Wei[2], Hongwei Xu[3], Ying Liu[3], Yong Meng[4], Ming-Qing Gao[4]*

[1]College of Life Sciences, Northwest University, Xi'an, China.

[2]Department of Pathology, Xi'an No.3 Hospital, the Affiliated Hospital of Northwest University, Xi'an, China.

[3]Basic Medical College, Qingdao University, Qingdao, China.

[4]School of Medicine, Northwest University, Xi'an, China.

*Corresponding author: gaomingqing@nwu.edu.cn.

Supplementary Materials

(Table S1 – S4 and Figure S1 – S3)

Table S1. Clinical parameters of the three breast cancer patients

Patients	Histology	WHO Grade	Her2 status	ER status	PR status	ki67	P53
1	IBC-NST	2	1+	80%+	70%+	15%+	+
2	IBC-NST	2	3+	-	-	40%	+
3	IBC-NST	2	2+	90%+	90%+	30%	Pos

WHO, World Health Organization; ER, estrogen receptor; Her2, human epidermal growth factor receptor 2; PR, progesterone receptor; IBC-NST, Invasive breast carcinoma of no special type.

Table S2. PCR primer sequence

Gene	Forward Primer	Reverse Primer
GAPDH	GGAGTCCACTGGCGTCTTCA	GTCATGAGTCCTTCCACGATACC
BMPR1B	GAGGATGACTCTGGGTTGCC	AGGCAGTGTAGGGTGTAGGT

Table S3. The specific information for each sample

patient	zone	High quality cells	mean reads/cell	total genes
1	tumor	11108	19727	35955
1	interface	12645	23938	36286
1	normal	13589	20801	35989
2	tumor	9597	38123	36618
2	interface	8539	32706	36680
2	normal	8960	36773	36821
3	tumor	8013	36323	36676
3	interface	8008	55136	37655
3	normal	8089	34588	36065

Table S4. Numbers of immune cells with immune hot and cold activities

Patients	Zones	cluster 0 (CD8+ T) hot	cluster 1 (CD4+ T) hot	cluster 2 (CD4+ T) cold	cluster 3 (Tregs) cold	cluster 4 (CD8+ T) hot	cluster 5 (CD8+ T) hot	cluster 6 (Tregs) cold	cluster 7 (CD8+ T) hot	cluster 8 (CD4+ T) hot	Total cell Number (hot)	Total cell Number (cold)
1	Normal	607	59	434	0	120	47	6	1	0	834	440
1	Interface	961	56	373	0	64	65	14	0	0	1146	387
1	Tumor	812	60	330	1	134	34	317	7	3	1050	648
2	Normal	73	35	75	1	60	60	20	4	3	235	96
2	Interface	20	985	33	174	381	94	507	73	127	1680	714
2	Tumor	15	63	46	1175	297	19	141	829	764	1987	1362
3	Normal	36	75	21	0	9	195	0	0	2	317	21
3	Interface	4	93	19	0	4	320	1	0	3	424	20
3	Tumor	37	207	138	2	138	312	88	7	5	706	228

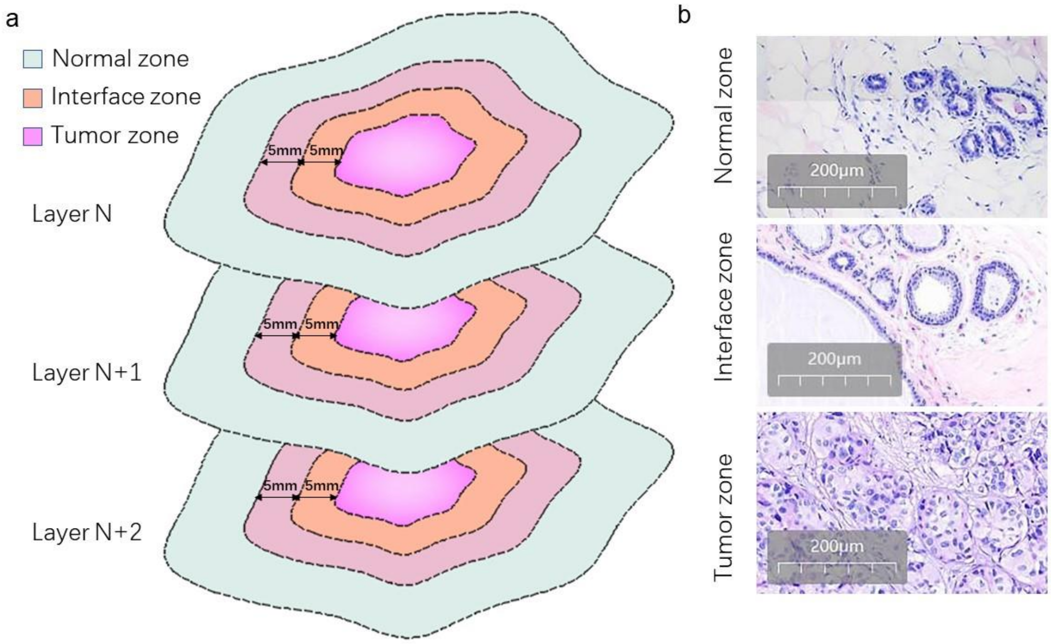

Figure S1. Identification and collection of representative samples. a Breast cancer tissues from the normal zone, interface zone and tumor zone were acquired by macroscopic dissection based on geographical mapping**.** Layer N, N+1, N+2 represented three continuous sections that have similar tumor boundary in the cutting surface. **b** Histological appearances of the normal, interface and tumor zone confirmed by hematoxylin and eosin (H&E) staining of representative tissues.

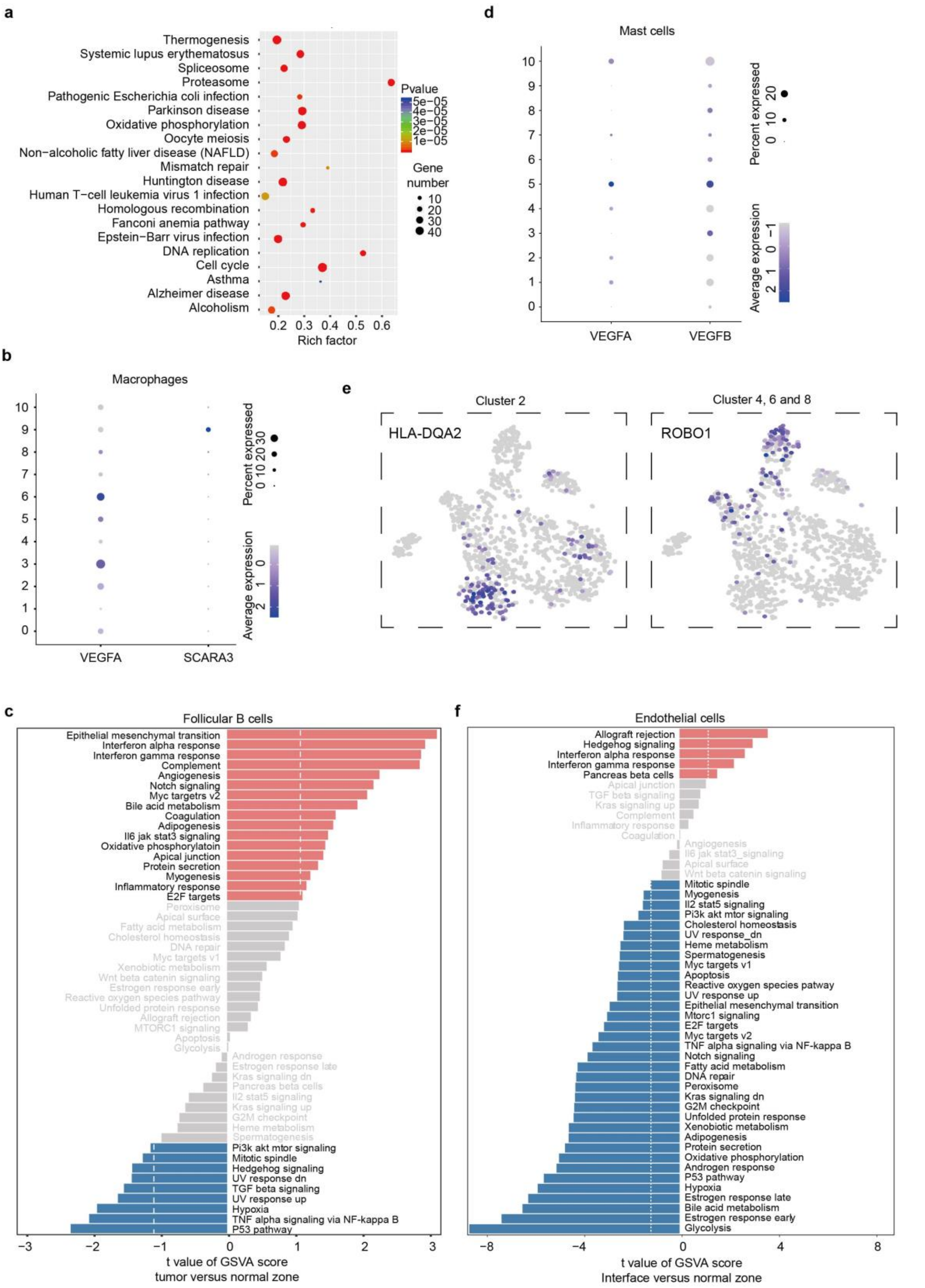

Figure S2. Gene expression and pathway analysis in stromal cells. a Kyoto Encyclopedia of Genes and Genomes (KEGG) pathway analyses of marker genes in TOP2A+ T cells. **b** Bubble plots displaying expressions of selected genes in macrophage clusters. **c** Differences in pathway activity of each cell scored by GSVA between follicular B cells isolated from normal and tumor zones. **d** Bubble plots displaying the expression of selected genes in mast cell clusters. **e** tSNE

plots of the specific gene expression of endothelial cell clusters. **f** Differences in pathway activity of each cell scored by GSVA between endothelial cells isolated from normal and interface zones.

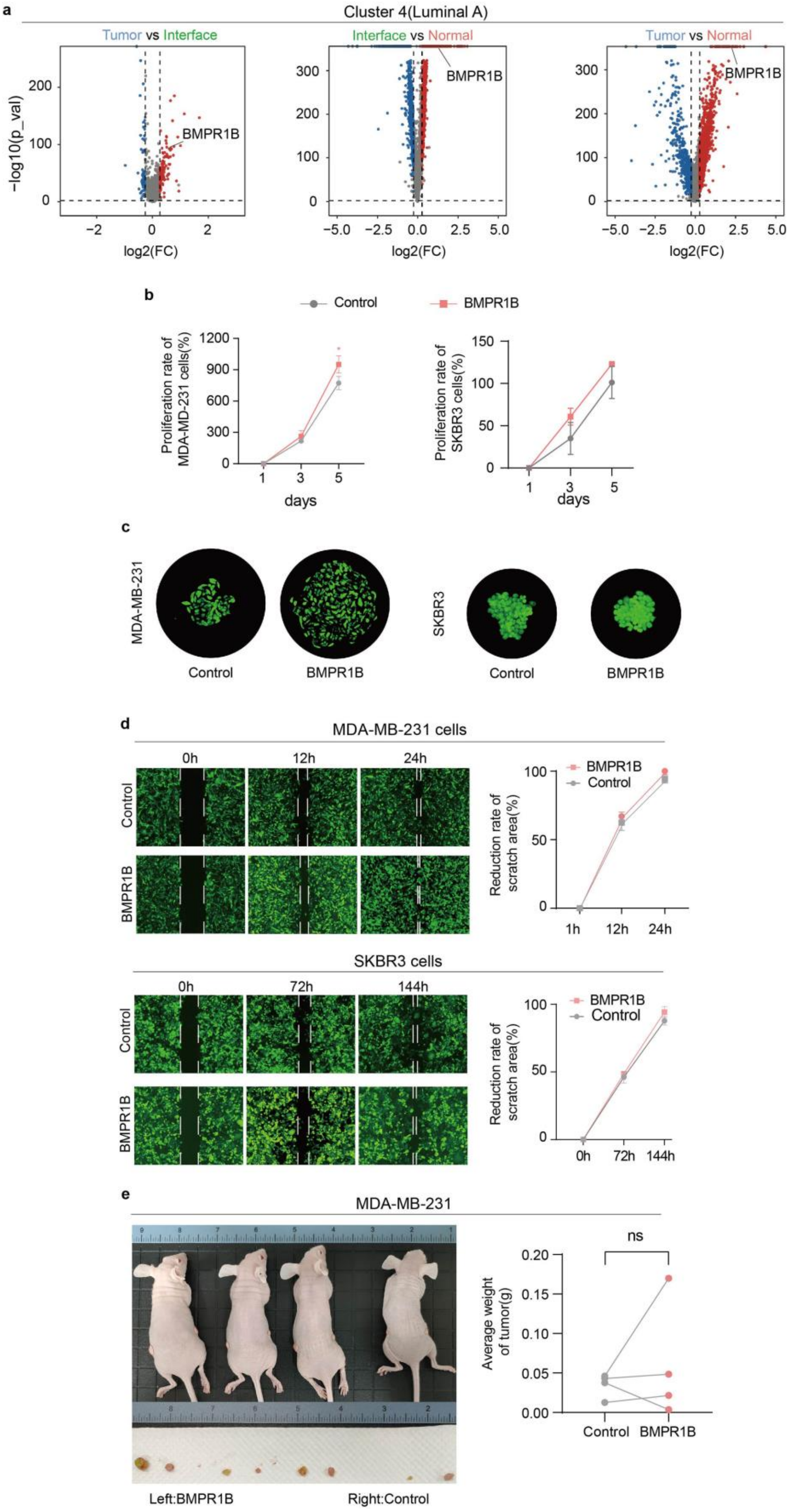

Figure S3. The expression and function of BMPR1B gene in epithelial cells. a Volcano plots of BMPR1B gene expression in cluster 4 from different zones here (left to right): Tumor zone versus interface zone, interface zone versus normal zone and tumor zone versus normal zone. **b** Line chart of cell proliferation measured at 450 nm. Cells with raw vector served as control group. **c** Photos of cell clones observed under a microscope. Cloning formation of BMPR1B-expressing MDA-MB-231 and SKBR3 cells versus matched cells with raw vectors. **d** Wound-healing assay of MDA-MB-231 and SKBR3 cells with or without BMPR1B overexpression. Left: representative images of BMPR1B-overexpressed cells and control cells at three time point after wounding. Right: quantification of the reduction rate of scratch area (mean±SD). **e** Photographs of BALB/cNj-Foxn1^{nu}/Gpt mice. (left) BMPR1B-overexpressed MDA-MB-231 cells and control cells with raw vector were respectively injected into subcutaneous location of left and right groin in nude mice. 8th week post-transplantation, mice were photographed, and tumors were removed surgically and weighed. (right) Statistical analysis of mice tumor weights, (n = 3, t test).
